# Subtle stressors—Strong responses. Consistent negative effects of avian blood parasites on phenotypic and demographic traits across songbirds

**DOI:** 10.1111/1365-2656.70106

**Published:** 2025-07-12

**Authors:** Marius Grabow, Sirkka Mang, Niels Blaum, Ralph Tiedemann, Viktoriia Radchuk, Stephanie Kramer‐Schadt

**Affiliations:** ^1^ Department of Ecological Dynamics Leibniz Institute for Zoo and Wildlife Research Berlin Germany; ^2^ Institute of Ecology, Technische Universität Berlin Berlin Germany; ^3^ Plant Ecology and Nature Conservation Universität Potsdam Potsdam Germany; ^4^ Systematic Zoology Universität Potsdam Potsdam Germany

**Keywords:** animal behaviour, avian blood parasites, demographic traits, meta‐analysis, pathogens, phenology, phenotypic traits, systematic review

## Abstract

1. Stressors that subtly yet persistently deplete energetic resources—such as heat, pollutants or parasites—are well studied in laboratory and clinical settings, where their physiological effects are often well understood, yet their influence on phenotypic and demographic traits in free‐living populations remains critically understudied.
2. A prominent example is pathogens and parasites that cause sublethal infections, often considered as relatively benign, particularly in species adapted to their presence. However, parasite‐induced effects on phenotypic and demographic traits are often inconsistent, leaving researchers uncertain about their impact and whether they have meaningful fitness consequences.
3. Here, we present a meta‐analysis evaluating the effects of avian blood parasites (*Plasmodium*, *Haemoproteus* and *Leucocytozoon*), a widespread and cosmopolitan stressor, on songbird species. Through a systematic review of 2473 publications, we identified 35 studies spanning 51 species and extracted 172 relevant effect sizes assessing host condition, phenology, reproduction and survival.
4. Our findings reveal consistent negative impacts: reductions in body condition, reproductive success and survival, along with delays in phenological events such as breeding. Furthermore, our findings revealed a critical research gap: despite the widespread study of avian blood parasites, only a limited number provide suitable effect sizes for assessing parasite impacts on demographic traits—let alone behavioural traits. This scarcity of data highlights the urgent need to understand pathogen‐induced effects on animal behaviour and demography, especially in the face of accelerating global change. We advocate for an integrative approach, combining behavioural, phenotypic, and demographic traits, to uncover the cascading consequences of parasitic infections on wild populations.

## Graphical abstract

Avian blood parasites are widespread and often considered benign, yet mounting evidence suggests they subtly impair host traits. This meta‐analysis is timely and necessary to quantify their true impact across systems, challenging assumptions and highlighting overlooked consequences for fitness and population dynamics.

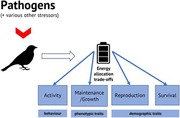

## 1 INTRODUCTION

Global change subjects individuals to multiple stressors, among them habitat degradation, pollution, climate change and diseases (Carrier‐Belleau et al., 2023; Chen et al., 2024; Daszak et al., 2000). Although it is generally accepted that stressors negatively affect individuals' Darwinian fitness (hereafter: fitness), sublethal effects on single traits have received less attention (Koltz et al., 2022; Shanebeck et al., 2022). Parasites, by definition, exploit their hosts by depleting resources, often at the expense of host health, survival, and reproduction (Poulin, 2011); therefore, they represent an ideal study system to determine whether energetically costly stressors lead to alterations in phenotypic and demographic traits. While sickness signs are evident during acute infection, with some individuals at risk of dying, a huge portion of wildlife disease goes unnoticed as hosts either do not exhibit clinical signs of sickness (i.e. subclinical infection) or persist in chronic states (Chrétien et al., 2023; Grenfell & Dobson, 2008). Yet, experimental studies on chronic or subclinical diseases often conclude that their effects on performance and condition are negligible, frequently reporting minimal impacts (e.g. Atkinson et al., 2001; Hahn et al., 2018; Palinauskas et al., 2008; Yorinks & Atkinson, 2000). Importantly, these studies are typically conducted under artificial conditions, where animals in enclosures are provided with food ad libitum—being spared of the energetic demands of searching for food while foraging. However, a few recent studies on single free‐ranging species have shown that subclinical infection can have severe demographic consequences (e.g. Asghar et al., 2015; Dadam et al., 2019; Grabow et al., 2024), challenging the general assumption of subclinical infections as being relatively benign. A critical research gap remains: understanding how general these findings are across populations of wild‐living species.

In this context, avian blood parasites (Haemosporidia) in songbirds serve as an ideal study system for investigating the effects of subclinical infection under natural conditions due to their cosmopolitan distribution across all continents except Antarctica and their status as a classical model system in host–pathogen research (Valkiunas, 2004). Avian blood parasite prevalence varies across geographic regions, landscapes and climates (Fecchio et al., 2021). They commonly reach high infection rates in songbirds, with over 40% of individuals infected on average (this study), and some study systems even reaching prevalences of up to 100% (e.g. De Amaral et al., 2023; Schumm et al., 2024). Haemosporidia are vector transmitted by blood‐sucking Diptera of various species like mosquitoes (Van Hemert et al., 2019). By infecting the red blood cells of hosts and feeding on haemoglobin, the parasites eventually destroy the red blood cells of hosts (note: Leucocytozoon can infect white blood cells; Valkiunas, 2004). During this primary phase of infection, the abundance of parasites in blood cells, that is parasitaemia, is typically high, potentially leading to a disrupted oxygen transport to organs, thereby diminishing the host's performance capacity (Valkiunas, 2004). During this acute phase, immediate behavioural reactions such as moving less to preserve energy can occur (Grabow et al., 2024; Mukhin et al., 2016; Yorinks & Atkinson, 2000), which may translate into effects on demography (Hawley & Altizer, 2011; McElroy & de Buron, 2014). Predicting the effects of avian blood parasites under natural conditions remains challenging, but recent research highlights the importance of trait‐based measurements as predictors of demographic change (Cerini et al., 2023; Clements et al., 2019; Clements & Ozgul, 2016; Pelletier et al., 2007). Quantifying the relative differences between infected and non‐infected individuals represents a promising approach to estimate the parasite‐induced impacts.

Cross‐sectional studies in wild populations, where some individuals are infected, provide the opportunity to investigate how infection status influences phenotypic and demographic traits. Phenotypic traits are plastic traits like body condition, which are shaped by individual behavioural decisions. Together, behavioural traits such as movement and phenotypic traits like body condition can lead to altered phenology, such as shifts in egg‐laying or spring arrival dates, ultimately impacting demographic traits like reproduction and survival (Figure S1). For example, infected individuals are commonly observed to reach stopover sites or breeding grounds later in the year than their non‐infected conspecifics (Asghar et al., 2011; Hegemann et al., 2018; López et al., 2013; Risely et al., 2018). While this may be related to alterations in migratory restlessness (‘Zugunruhe’; Kelly et al., 2020) or to alterations in the diet of infected individuals (‘adaptive feeding’; Jiménez‐Gallardo et al., 2025), it usually negatively affects the infected individual, as delays are deviations from optimal migration strategy (Alerstam et al., 2003; Hall et al., 2016, 2022). Indeed, delayed arrivals to breeding grounds often result in fewer nesting and mating opportunities, lower resource availability, and reduced offspring condition (e.g. Costa et al., 2021). Therefore, phenology may serve as a flexible proxy to predict impairments caused by avian blood parasites and that may be linked to fitness.

To date, studies investigating alterations of phenotypic and demographic traits in infected individuals have yielded inconclusive results. For example, severe declines in survival were observed in Hawaii (USA) in various forest birds after the introduction of avian malaria (caused by *Plasmodium relictum*) and its vector *Culex quinquefasciatus*. On the continental mainland, such pronounced effects were traditionally not expected, because hosts have a long‐standing co‐evolutionary history with these parasites (Palinauskas et al., 2008). Indeed, several studies reported minimal, if any, consequences on host survival (Bensch et al., 2007; Hammers et al., 2016; Zylberberg et al., 2015). Likewise, inconsistent results were reported for reproduction (Kilpatrick et al., 2006; Marzal, Reviriego, et al., 2013; Zylberberg et al., 2015), body condition (Bichet et al., 2020; Chakarov & Blanco, 2021; De La Torre et al., 2020) and phenology (Emmenegger et al., 2018, 2021; Hegemann et al., 2018; Kelly et al., 2020).

However, this traditional notion of avian blood parasites—shared co‐evolutionary history reduces fitness losses—has recently faced opposition. In a seminal paper, Asghar et al. (2015) studied great reed warblers and confirmed that even low‐level chronic infection significantly reduces lifespan. The study made the important point that chronic or subclinical infection may cause subtle effects that accumulate and reduce fitness (Asghar et al., 2015), regardless of whether immune systems have evolved defence mechanisms. At the same time, the development of more sensitive molecular detection methods such as polymerase chain reaction (PCR), which can simultaneously detect multiple parasite genera (Hellgren et al., 2004), increased the number of studies considering avian blood parasites (Clark et al., 2014). These studies often confirmed phenotypic and demographic responses even in species that share evolutionary history with the pathogens (Asghar et al., 2015; Marzal et al., 2008; Marzal, Reviriego, et al., 2013; Merino et al., 2000). However, clear evidence of systematic patterns remains elusive. Uncertainty remains as to which phenotypic and demographic traits are most vulnerable and whether reproduction or survival is affected most. This knowledge may be particularly important in times of rapid environmental shifts, where subtle stressors of several kinds (including pathogens) may accumulate in their effects on a population (Carrier‐Belleau et al., 2023; Gamelon et al., 2023; Scheffer et al., 2009).

We conducted a systematic review to assess how *Plasmodium*, *Haemoproteus* and *Leucocytozoon* affected phenotypic traits such as host condition, movement behaviour, and phenology, and demographic traits such as reproduction and survival (Figure S1). Analysing the blood parasite genera collectively, as is standard in molecular studies (Hellgren et al., 2004), provides a broader understanding of their shared impacts on hosts. We expected widespread impairments across phenotypic and demographic traits, reflecting reduced host performance capacity. Phenotypic traits, particularly highly plastic ones like body condition, are tightly linked to infection‐induced behavioural changes, such as altered movement, and tend to respond more rapidly and severely to impairments. For instance, foraging impairments (behaviour) are likely to result in immediate reductions in body condition, a highly plastic trait. Body condition is a standard measurement in ecological studies (e.g. Bairlain et al., 1995; Kaiser, 1993), and we anticipated more studies addressing this trait category. Conversely, we expected fewer studies on survival, as long‐term datasets required for such investigations are scarce.

## 2 METHODS

### 2.1 Systematic literature search

We performed a systematic literature search to evaluate the responses of phenotypic and demographic traits of songbirds to infections by avian blood parasites—*Plasmodium*, *Haemoproteus* and *Leucocytozoon*. We categorised trait responses into four trait categories: (1) host condition (here defined broadly, incorporating morphological traits such as body condition, growth and physiological traits such as oxidative stress levels; see Table S2 and Discussion S2), (2) phenology (e.g. timing of breeding or migration), (3) reproduction (e.g. fledging success) and (4) survival. We also searched for studies addressing behaviour (Figure S1), particularly movement behaviour (see search string below), but were unable to include this trait category as no relevant studies were found (but see: Grabow et al. (2024)). To identify the studies satisfying the above‐mentioned criteria, we searched the Web of Knowledge (search conducted on 26.10.2024) combining the following keywords:

((ALL = ((songbird* OR passerine* OR Passeriform* OR bird* OR avian)))
for identification of host species.
AND ALL = ((plasmod* OR haemoprot* OR hemo* OR haemosporid* OR blood parasit* OR “leukocytozoonosis” OR leucocytozo* OR “haemoproteoses” OR “avian malaria”) AND (“prevalence” OR infect* OR infest* OR “disease” OR “chronic”))).
for targeting avian blood parasites of all three genera.
AND ALL = ((move* OR dispers* OR migrat* OR distribut* OR forag* OR “fitness” OR surviv* OR behav* OR pheno* OR morph* OR “mortality” OR reproduc* OR “offspring” OR “clutch” OR breed* OR “egg laying date” OR fledg* OR hatch* OR “body mass” OR “body length” OR “body condition” OR “wing length” OR “size” OR “weight” OR “tarsus”)).
for targeting phenotypic and demographic trait categories. For haemoprot*, haemosporid* and haemoproteos*, we also checked for the alternative writing hemoprot *, hemosporid* and hemoproteos*.

**FIGURE 1:**
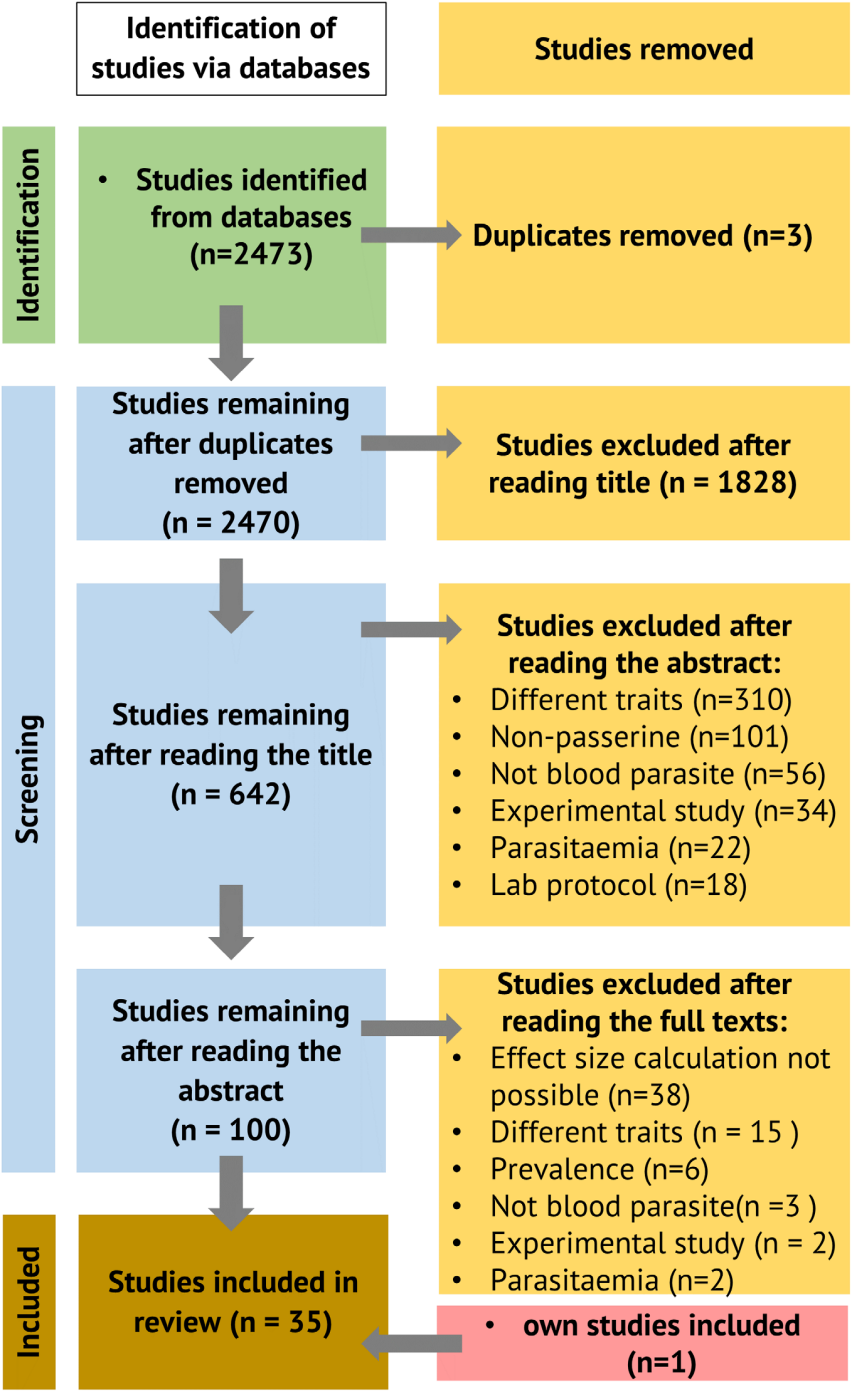
PRISMA flow diagram for illustrating the systematic literature review following Page et al. (2021). Inclusion criteria: (1) The study focussed on songbirds (Passeriformes); (2) individuals were not held in captivity; (3) there was no experimental manipulation of infection status; (4) Infection status is categorical (infected/non‐infected).

We used the Preferred Reporting Items for Systematic reviews and Meta‐Analyses framework (PRISMA; Page et al., 2021) for reporting our workflow (Figure 1). The literature search returned 2473 publications, 100 of which we retained after reading the abstracts. We included one recent study that was published after the literature search was conducted (Grabow et al., 2024). We removed duplicate entries when publications reported on the same study system, retaining only the earliest publication. While reading the abstracts, we removed studies conducted on orders other than songbirds (Passeriformes) to streamline our analyses towards phylogenetically similar species and excluding non‐adapted species that could have more severe impacts by infection. For example, some penguin species have naturally never encountered avian malaria, thus have limited immune defence (naïve hosts), often resulting in death in zoos (Hernandez‐Colina et al., 2021; Ings & Denk, 2022). Further, we removed studies conducted on captive or domestic birds, because birds in captivity typically do not forage naturally. Given that experimental conditions control for all other factors than the one under considerations, this manipulation does not reflect all natural processes (such as foraging, predator avoidance, etc.). Likewise, we removed studies that used medication to treat infection or infected individuals experimentally, as these experiments are typically conducted in enclosures, streamlining our analysis towards natural haemosporidian infections. Furthermore, we excluded studies that used parasitaemia levels (infection intensity) as the independent variable instead of a categorical infection status (infected / non‐infected), because our preliminary literature exploration showed that the number of such studies was rather small (Figure 1). Our study did not require ethical approval.

We assembled our data set by extracting data wherever possible and contacted authors in case of missing data to calculate effect sizes. Additionally, we extracted the geographical coordinates of the study location, the bird species, the number of sampled birds and the number of studied locations, the prevalence of blood infection and co‐infection with other blood parasites or the number of infected birds, and the laboratory methods that were used to detect the parasites. Eventually, 35 publications (Agh et al., 2019; Arizaga et al., 2009; Asghar et al., 2015; Bennett et al., 1988; Coon et al., 2016; Coon & Martin, 2014; Davidar & Morton, 2006; Figuerola et al., 1999; Garvin et al., 2006; Grabow et al., 2024; Hatchwell et al., 2001; Hegemann et al., 2018; Hernández et al., 2020; Kilpatrick et al., 2006; Kubacka et al., 2019; Magallanes et al., 2017; Marzal et al., 2008; Marzal, Asghar, et al., 2013; Marzal, Reviriego, et al., 2013; Mora‐Rubio et al., 2024; Muriel et al., 2023; Norte et al., 2009; Oppliger et al., 1997; Ots & Horak, 1996; Piersma & van der Velde, 2012; Poblete et al., 2024; Podmokla et al., 2014, 2017; Rätti et al., 1993; Romano et al., 2019; Sanz et al., 2001; Schrader et al., 2003; Shurulinkov et al., 2012; Votypka et al., 2003; Weatherhead & Bennett, 1992; Yusupova et al., 2023) contained the data necessary to calculate effect sizes for 172 phenotypic and demographic traits, as some publications reported more than one trait (Table S2). Data on phenotypic traits was typically collected at the same time than obtaining infection statuses, while data on demographic traits require long‐term data and was typically assessed after infection statuses were assigned (e.g. survival is typically estimated between two consecutive years).

### 2.2 Meta‐analysis

To analyse the general trait responses across different host species, parasite species, and study locations, we conducted a meta‐analysis employing a multilevel mixed‐effects model in a Bayesian framework. First, we quantified effect sizes using Hedge's *g* (Hedges, 1981), derived from standardised mean differences (SMD):

(1) $$g=\frac{{\bar{x}}_{1}-{\bar{x}}_{2}\;}{s_{\text{pooled}}}\times \left(1-\frac{3}{4N-9}\right)$$

where ${\bar{x}}_{1}$ and ${\bar{x}}_{2}$ are the means of non‐infected and infected individuals, respectively. N is the combined sample size of both groups, and $s_{\text{pooled}}$ is the combined standard deviation, calculated as:

(2) $$s_{\text{pooled}}=\sqrt{\frac{\left(n_{1}-1\right)\;s_{1}^{2}+\left(n_{2}-1\right)s_{2}^{2}}{n_{1}+n_{2}-2}}$$

where $n_{1}$ and $n_{2}$ represent sample sizes, and $s_{1}$ and $s_{2}$ standard deviations of the respective groups. For effect size calculation, we prioritised using mean and standard error (SE) or standard deviation (SD) reported in the original studies, whenever possible, and only used values such as *F*‐values from ANOVA results, *t*‐values, or *χ* ^2^ results when raw data or mean values were unavailable. We used the *esc* package (Lüdecke, 2018) in R 4.3.3 (R Core Team, 2024) to calculate effect sizes, and followed Koricheva et al. (2013) if effect sizes had to be calculated from *F*‐values (*n* = 18), *t*‐values (*n* = 1) or chi‐square (*n* = 13) results. We calculated effect sizes for the trait categories by using infected individuals as the treatment group, that is, negative values in the effect sizes for condition, survival, or reproduction indicate decreased trait values such as lower body conditions, decreased survival, or fewer nestlings, respectively. Given that phenology refers to dates, negative effect sizes for this trait category describe earlier phenological events, and positive values indicate delays in phenological events compared to uninfected individuals.

We fitted a Bayesian hierarchical regression with the *brms* package (Bürkner, 2017). Specifically, we fitted a model with trait category as a fixed effect, while accounting for heterogeneity across species, study locations and publications by including random intercepts for bird species, study location and reference, respectively. We used a Gaussian family to model effect sizes (Hedge's *g*) and incorporated the reported standard error of effect sizes:

(3) $$g_{i,j,k}\sim N\left(\mu_{i,j,k},\sigma_{i}^{2}\right)$$

where:

(4) $$\mu_{i,j,k}=\beta_{1}{\text{Trait}}_{i,j,k}+\alpha_{\text{species}\left[i\right]}+\alpha_{\text{study location}\left[j\right]}+\alpha_{\text{publication}\left[k\right]}+\varepsilon_{i,j,k}$$

with $g_{i,j,k}$ representing the effect sizes (Hedges' *g*) for species i, study location j and publication k, $\beta_{1}$ being the fixed effect of the trait category for species i, study location j and publication k, and $\alpha \sim N\left(0,r^{2}\right)$ the random intercept for species *i*, study location *j* and publication *k* drawn from a Gaussian distribution with mean zero and *r* ^2^ and *ε*_*ijk*_~*N*(0, *σ*2), capturing residual variance.

This model was run with four chains for a total of 8000 iterations, with a burn‐in period of 2000 samples.

To evaluate whether phylogenetic relationships among species contributed to variation in effect sizes, we initially fitted a random‐intercept‐only model with a variance–covariance matrix derived from a species‐level phylogeny. However, this model exhibited high uncertainty in the phylogenetic variance estimate (95% CI: 0.01–0.89) and divergent transitions, suggesting that species‐level variation was weakly structured by evolutionary history. Given that study‐ and site‐level heterogeneity were of similar or greater magnitude as species‐level variance, we opted for a simpler model structure that excluded phylogenetic covariance while retaining species as a random intercept. Between‐study heterogeneity was assessed by partitioning variance across hierarchical levels, computing posterior distributions for the variance components of each factor. All reported values represent posterior means with 95% credible intervals (CI).

### 2.3 Sensitivity analysis

To mitigate potential biases from influential data points due to large sample sizes or extreme effect sizes, we assessed data influence using Pareto 𝑘 values from leave‐one‐out cross‐validation (LOO; Vehtari et al., 2017). We considered points with 𝑘 > 0.7 as influential, potentially biasing our results (Figure S3). In addition, we tested if the exclusion of the Hawaiian study system—distinct due to its lack of co‐evolutionary history—would yield similarly robust estimates (Table S4). Therefore, we re‐fitted models using exact LOO cross‐validation to obtain more robust estimates, that is one additional model‐fit per influential effect size 𝑘 > 0.7. To address potential publication biases, we followed the approach suggested by Nakagawa et al. (2022): we fitted a multi‐moderator meta‐regression, including the square root of the inverse effective sample size and publication year as fixed effects. This approach helped to mitigate the influence of studies with smaller sample sizes with disproportionately large effect sizes relative to sample size. Additionally, it allowed us to investigate potential temporal trends in effect sizes, ensuring that observed outcomes were not confounded by changes in effect magnitude over time. Finally, we also plotted meta‐analytic residuals against precision (inverse standard error) and inspected funnel plots for asymmetries.

## 3 RESULTS

### 3.1 Systematic literature search

**FIGURE 2:**
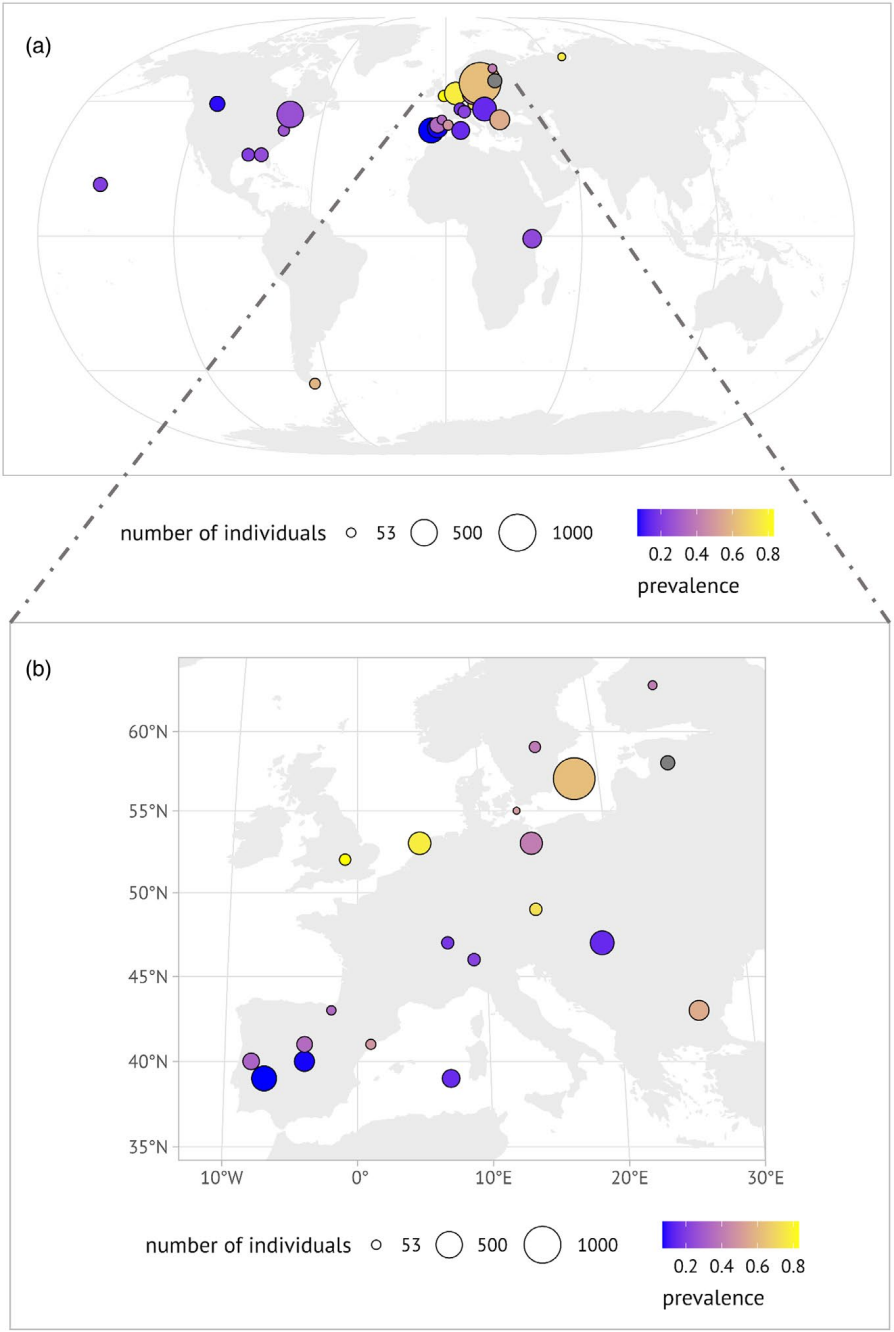
Overview map of included studies. Upper panel (a): Study locations (per reference) with included effect sizes. Point sizes indicate the number of individuals included in analyses; colour gradient shows the overall infection prevalence reported in the study. Lower panel (b): Study locations in Europe, with point sizes indicating the number of individuals included in analyses; colour gradient shows the overall infection prevalence reported in the study.

The dataset consisted of captures of 51 different passerine species in 18 countries, most of which were in Europe (Figure 2a,b). The sample sizes were on average 121.95 ± 149.28 (mean ± SD) per publication. Prevalence varied between studies, with a mean prevalence of any blood parasite infection being 45% ± 22% (mean ± SD), with a minimum 1.51% and a maximum 85.9% of individuals being infected. We calculated 172 effect sizes for trait responses of the 35 retained publications. We calculated more effect sizes than we had publications because some studies reported data for several trait responses or defined subgroups such as adults and juveniles. Publications contributed between 1 and 27 (mean: 4.91) effect sizes to our analysis.

### 3.2 Blood parasite effects on phenotypic traits and demographic traits

**FIGURE 3:**
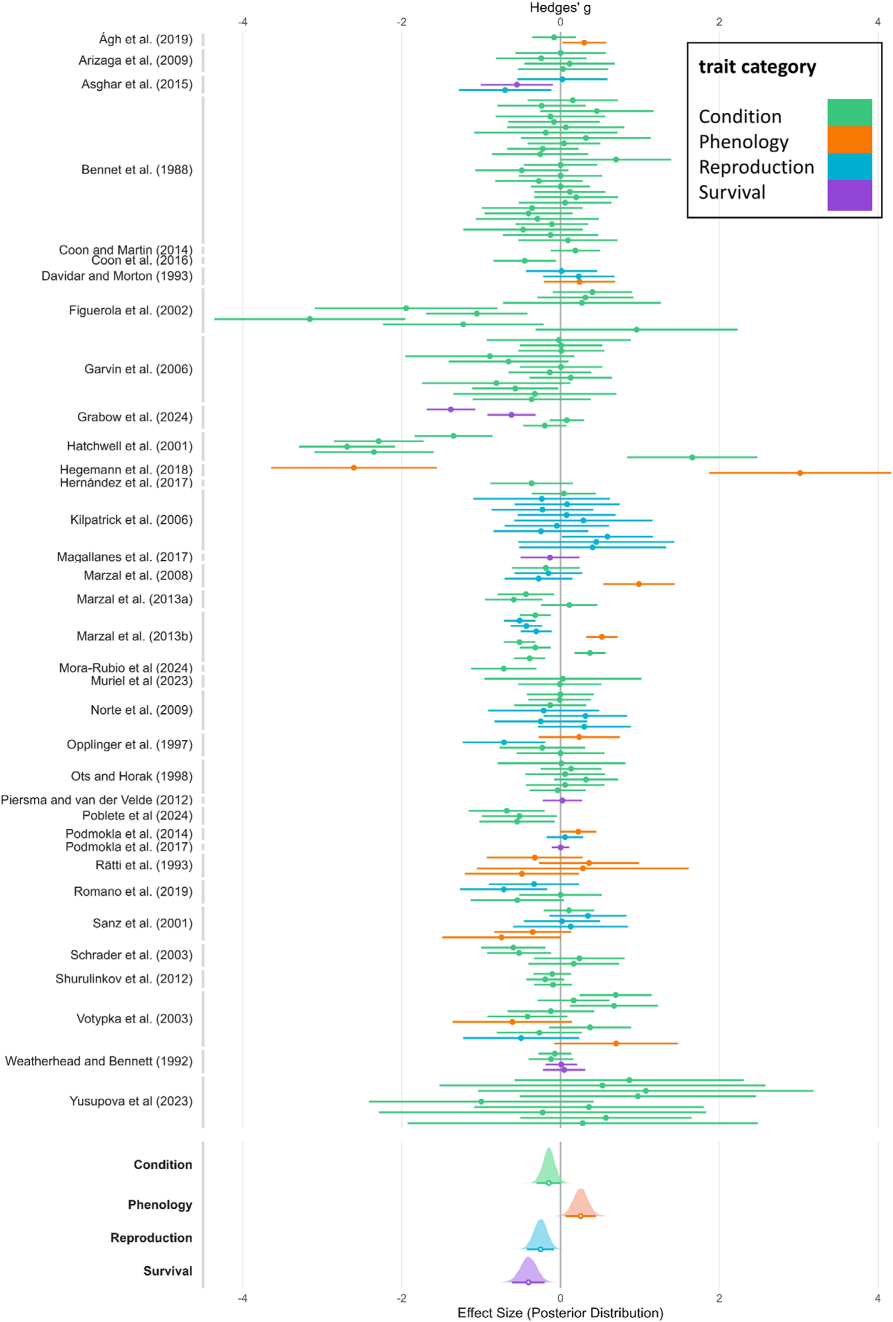
Forest‐plot with effect sizes (calculated as Hedge's *g*), grouped by reference (left), coloured by trait category (see legend). Each bar represents a trait category, with various studies reporting multiple effect sizes per trait category (e.g. because they studied multiple species or multiple traits within the same trait category). Posterior distributions (bottom) show effect sizes per trait category (point = mean, bar = 95% credible interval) after application of Bayesian multilevel meta‐regression.

In the Bayesian multilevel meta‐analysis, all chains converged, indicated by Rhat values of one (Gelman & Rubin, 1992). Across studies (Figure 3), our results indicate a decrease in body condition (i.e. any decrease of morphological trait measures; see Table S2) for infected individuals (Hedge's *g*: −0.15, CI 95%: [−0.30, −0.00]), as the credible interval did not overlap zero. Similarly, reproductive success (Hedge's g: −0.25, CI 95%: [−0.42, −0.08]) and survival (Hedge's *g*: −0.40, CI 95%: [−0.61, −0.20]) were lower in infected individuals. For phenology, we found increased effect sizes for infected individuals (Hedge's *g*: 0.25, CI 95%: [0.06, 0.44]), suggesting that an infection is associated with a shift in timing to later dates. Marginal Bayesian *R* ^2^ was 0.043 (CI95%: [0.025, 0.065]), conditional *R* ^2^ was 0.236 (CI95%: [0.170, 0.314]). Among the latter, the largest part of the variance was explained by reference (39.30%, CI95%: [0.02, 94.13]), followed by study location (28.57%, CI95%: [0.00, 83.56]) and species identity (13.78%, CI95%: [0.00, 72.41]).

### 3.3 Sensitivity analysis

After applying LOO cross‐validation, we found no highly influential studies (Figure S3). Furthermore, we found no asymmetry when inspecting funnel plots, as the measurement of the certainty of the effect size estimates versus the effect sizes (Figures S5–S8). Our multi‐moderator meta‐regression analysis revealed no evidence of biases related to smaller studies, which typically exhibit larger standard errors and consequently higher values of the square root of the inverse effective sample size (Figure S9). Similarly, the analysis using the year of publication as a moderator did not find an indication of time‐lag bias (Figure S10).

## 4 DISCUSSION

We analysed phenotypic and demographic traits of songbirds infected or non‐infected with avian blood parasites. We identified consistent negative effects of infection across multiple traits: reduced host condition, delayed phenology, diminished reproduction and survival. Our findings add to the growing evidence that avian blood parasites exert substantial physiological costs. While severe impacts are well‐documented in specific systems, such as Hawaiian forest birds infected with avian malaria (Atkinson et al., 1995; van Riper III et al., 1986), our results generalise these effects across songbird species, beyond acute infection, host specificity or geographic regions. Despite occasional reports of neutral or even positive effects of parasitism, our study aligns with the core principle of parasitism: Parasites drain energetic host resources or induce structural or functional damage, leaving fewer resources for fitness‐related functions (Sheldon & Verhulst, 1996).

A major limitation—simultaneously a key finding—is that demographic traits were underrepresented, with few studies reporting effects at all and with generally small effect sizes. Nonetheless, Hedges' *g*, as a standardised effect size that expresses differences among groups in units of standard deviation (SD), requires biological contextualisation. To illustrate the real‐world relevance of seemingly small effects, we highlight two empirical studies from our dataset whose effect sizes align with our meta‐analytic estimates. Asghar et al. (2015) found that non‐infected great reed warblers lived 2.53 years (±0.26 SD) on average, compared to 1.69 years (±0.15 SD) in infected individuals, representing a reduction of 33% in lifespan. While this effect size (Hedges' *g*: −0.54 ± 0.23 SD) may be considered small to moderate by conventional standards (Cohen, 2013; but see Durlak, 2009; Volker, 2006), this decline has profound implications for population dynamics, and dismissing it would overlook substantial ecological costs. Similarly, Marzal et al. (2008) found an approximately 10% reduction in fledgling numbers in single‐infected individuals (Hedges' *g*: −0.27 ± 0.21 SD). Though considered small statistically, such effects can meaningfully impact population viability. These cases underscore the need to evaluate biological relevance beyond conventional statistical thresholds.

Unarguably, each of the analysed trait categories—(1) host condition, (2) phenology, (3) reproduction, and (4) survival—is closely tied to both host activity and fitness. Behavioural alterations are likely to occur first in response to infection, preceding measurable changes in phenotypic traits and potential fitness changes (Ezenwa et al., 2016; McElroy & de Buron, 2014). Interestingly, pathogen‐induced alterations in behaviour had to be excluded, as no studies reported effect sizes in the required format, despite strong evidence that these traits are highly responsive to infection (Grabow et al., 2024, 2025; Kelly et al., 2020; Mukhin et al., 2016; Schoepf et al., 2022). Host movement, typically impaired by infection (Risely et al., 2018), is particularly important, as it may serve as an early indicator (see Figure S1) of pending impairments in other traits (McElroy & de Buron, 2014). For instance, body condition—a highly variable phenotypic trait—is directly shaped by recent foraging behaviour (Clark, 1979; Krause et al., 2017). The absence of movement‐related behavioural traits in our dataset underscores a critical research gap: pathogen‐induced alterations in (movement) behaviour remain understudied (Binning et al., 2017). The following discussion outlines how incorporating behavioural traits—particularly those with rapid response times—can shed light on early‐stage infection effects and their cascading consequences on other traits (Figure S1).

1. Host body condition. We found that across the studies, the effect size was the smallest for body condition, with the highest number of collected estimates. This aligns with the understanding that body condition is inherently variable, with variation of 25% in body weight within a day in small animals (Clark, 1979; Krause et al., 2017). Likewise, the small effect size is in agreement with experimental studies that performed medication on infected birds and did not necessarily point to changes in host body condition (Schoenle et al., 2017; Yorinks & Atkinson, 2000). Impaired body condition may directly reflect altered immune responses or indirectly result from infection‐induced behavioural changes. Behavioural alterations may manifest either as adaptive sickness behaviours or as general reductions in host performance due to compromised health (Binning et al., 2017; McElroy & de Buron, 2014).
2. Phenology. We found that phenological events like egg laying consistently occurred later in infected individuals, disrupting the evolved timing of migration (Hall et al., 2016). Previous research has demonstrated that higher infection intensities and compromised immune responses correlate with delayed arrivals at stopover sites, suggesting that infection reduced available energy for migratory movements (Emmenegger et al., 2018). Such delays during migration have been repeatedly documented (Asghar et al., 2011; Moller et al., 2004; Rätti et al., 1993), consistent with the understanding that migration represents one of the most energetically demanding phases in hosts' annual cycles (Hall et al., 2022). Another possible—not mutually exclusive—explanation is that energetic demands increase in hosts infected by sublethal infections (Hicks et al., 2018), forcing birds to spend more time refuelling or selecting energy‐rich habitats during stopovers (Jiménez‐Gallardo et al., 2025).
3. Reproduction. Avian blood parasites negatively affected host reproduction. Severe reproductive impacts are expected during the acute phase of infection (Schoepf et al., 2022), often aligning with the period of reproduction (Applegate, 1971; Cosgrove et al., 2008). However, Asghar et al. (2011) confirmed that negative impacts on reproduction are also found under chronic infection. The observed impairments—again—may stem from various underlying mechanisms. In studies that measure clutch sizes or egg weights, impairments suggest that chronic stressors may translate into reduced investments prior to hatching (e.g. Marzal et al., 2005; Merilä & Andersson, 1999). Parasite infection may also influence mate choice (Hamilton & Zuk, 1982), potentially to the point of completely giving up reproduction, although this aspect remains largely underexplored in free‐ranging populations due to the difficulty of measurement. Furthermore, the infection status in adults may lead to impaired offspring conditions. For example, in a medication experiment in blue tits (*Cyanistes caerulus*), Merino et al. (2000) confirmed that infected parents had reduced parental care capacity, with juveniles paying the cost of infection. Reductions in parental care activities were also confirmed in red‐winged blackbirds (*Agelaius phoeniceus*) infected with avian blood parasites (Schoepf et al., 2022).
4. Survival. Survival was a demographic trait most strongly impacted by the infection. Interestingly, despite survival having direct implications for fitness, we found very few studies focusing on the effects of blood parasites on bird survival. This may reflect the difficulty of assessing survival in wild birds, which requires either repeated monitoring (Pigeault et al., 2018) or analysis of carcasses, which are rarely found in free‐ranging populations (Wobeser, 2006). Our results emphasise that negative effects on plastic traits such as body condition and phenology may accumulate, intensifying impacts on demographic traits. For instance, reductions in movement behaviour can decrease body condition (Grabow et al., 2025) and delay migration due to insufficient fat reserves (Guglielmo, 2018). This could subsequently delay the breeding season and ultimately reduce survival probabilities for fledgelings. Consistent with our results, experimental studies also frequently reported reduced survival (e.g. van Riper III et al., 1986), both in species that show no co‐evolutionary history with the pathogens and those in which pathogens are endemic (Asghar et al., 2015; Dadam et al., 2019; Grabow et al., 2024). Although linking mortality to behaviour is less straightforward, growing evidence suggests that infected hosts face heightened predation risk (Adelman et al., 2017) or depletion of resources (Smith et al., 2005).

It is noteworthy that, although we observed negative effects across all phenotypic and demographic trait categories, this does not necessarily equate to absolute fitness losses, as declines in one of the traits may trade‐off against increases in others (Stearns, 1992). For example, individuals with increased risks of mortality could increase compensatory investments in reproduction (Culina et al., 2019). Such ‘terminal investment’, an increased investment in immediate reproduction at the cost of survival, allows propagating genes despite reduced survival prospects (Velando et al., 2006; Williams, 1966). Indeed, Pigeault et al. (2018) documented such increased reproductive output at the cost of survival in great tits (*Parus major*) infected with haemosporidians. The lack of studies conducted on trade‐offs calls for future studies to focus more thoroughly on those. Our dataset and the methodological constraints do not allow us to make inferences about such demographic trade‐offs, because only one of the included studies (Asghar et al., 2015) had investigated both demographic trait categories at the same time.

A further important limitation of our study is the geographic bias in the published data, which was predominantly collected in Europe and North America, despite our aim to investigate global impacts of haemosporidian infection. Despite being common in ecological research, this bias has important implications for the generalisability of results (Hughes et al., 2021). In the case of avian blood parasites, focusing on the Northern Hemisphere often restricts analyses to breeding periods, while host responses during the overwintering period remain largely unknown (but see e.g.: Dunn et al., 2013). Similarly, we have chosen a broad trait categorisation, inevitably introducing some heterogeneity—particularly within ‘condition’ (see Discussion S1).

We studied infection status instead of intensity (parasitaemia). Such binary categorisation can oversimplify results (e.g. Hahn et al., 2018; Schoenle et al., 2017), since severe effects are expected during the acute infection phases with active red blood cell destruction (Valkiunas, 2004), while chronic stages often go unnoticed (Asghar et al., 2011). Although information on infection intensity would be valuable, repeatedly measuring parasitaemia in wild populations is challenging due to rapid parasite replication and frequent fluctuations between chronic and acute states. Single‐time sampling rarely captures these fluctuations, making it difficult to connect parasitaemia at sampling to subsequent behavioural outcomes. For example, individuals that were sampled early in the season and showed low parasitaemia might later experience delayed migration if their infection intensity increases. Nonetheless, our focus on infection status in wild populations led to robust findings, as severely infected individuals are typically underrepresented (Binning et al., 2022). This likely results in our estimates being conservative, as individuals with the most severe infections may experience higher mortality or behavioural impairments that prevent them from being detected in the first place.

In conclusion, we demonstrate that avian blood parasites exert generalisable negative effects on phenotypic and demographic traits, likely by reducing individual performance capacity. As such, physiological impacts are expected to occur first, with alterations in activity and behaviour following, which then impair such phenotypic traits as host body condition. Although less pronounced and less often observed, our study shows that demographic traits are impacted as well, potentially following the effects on phenotypic traits that individuals face due to infection. Hence, we advocate for studying the short‐term effects of pathogens and parasites from a behavioural perspective, incorporating plastic phenotypic traits including movement behaviour alongside demographic traits. To conserve biodiversity, it is essential for behavioural ecologists and population ecologists to combine their efforts to better understand how even subtle stressors affect wild animals and populations.

## AUTHOR CONTRIBUTIONS

Marius Grabow, Sirkka Mang, Viktoriia Radchuk, and Stephanie Kramer‐Schadt designed the study; Marius Grabow and Sirkka Mang performed the literature search; Marius Grabow, Viktoriia Radchuk, and Stephanie Kramer‐Schadt analysed the data; Niels Blaum, Ralph Tiedemann, Viktoriia Radchuk, and Stephanie Kramer‐Schadt provided project supervision; Marius Grabow led the writing of the manuscript; all authors edited the manuscript and made valuable scientific contributions throughout the writing process.

## FUNDING INFORMATION

M.G. was supported by the German Research Foundation (DFG) Research Training Group ‘BioMove’ (DFG‐GRK 2118/2). N.B., R.T., V.R. and S.K.‐S. are associated with the DFG Research Training Group ‘BioMove’ (DFG‐GRK 2118/1 and 2).

## CONFLICT OF INTEREST STATEMENT

The authors declare no competing interests.

## Supporting information


**Figure S1.** Conceptual framework illustrating the relationships between phenotypic and demographic traits, and their feedback to fitness.
**Table S2.** Overview of phenotypic and demographic trait categories and the detailed traits that were included in each category. Sample size (*n*) describes number of effect sizes calculated for each trait category.
**Table S4.** Model estimates from the original manuscript compared to estimates without the Hawaiian study system, which is known to differ from many other study systems due to the lack of evolutionary history between host and parasite.
**Figure S3.** Identification of influential studies using Pareto *k*. Dashed orange line (*k* = 0.7) indicates influential studies, dashed red line (*k* = 1) indicates highly influential studies.
**Figure S5.** Funnel plot for host condition. Each point represents one effect size, coloured by study.
**Figure S6.** Funnel plot for host survival. Each point represents one effect size, coloured by study.
**Figure S7.** Funnel plot for host reproduction. Each point represents one effect size, coloured by study.
**Figure S8.** Funnel plot for phenology. Each point represents one effect size, coloured by study.
**Figure S9.** Relationship between inverse effective sample size and effect sizes. Each point represents an individual study, with colours differentiating study categories. Blue line and shaded are represent predicted effect size and 95% CI. The symmetrical horizontal distribution indicates minimal bias regarding study size.
**Figure S10.** Relationship between study year and effect sizes. Each point represents an individual study, with colours differentiating study categories. Blue line and shaded are represent predicted effect size and 95% CI. The symmetrical horizontal distribution indicates minimal bias regarding study year.

## Data Availability

Data available from the Zenodo digital repository https://zenodo.org/records/14420924 (Grabow, 2024).

## References

[jane70106-bib-0001] Adelman, J. S. , Mayer, C. , & Hawley, D. M. (2017). Infection reduces anti‐predator behaviors in house finches. Journal of Avian Biology, 48(4), 519–528. 10.1111/jav.01058 29242677 PMC5724792

[jane70106-bib-0002] Agh, N. , Piross, I. , Majoros, G. , Csörgo, T. , & Szöllosi, E. (2019). Malaria infection status of European Robins seems to associate with timing of autumn migration but not with actual condition. Parasitology, 146(6), 814–820. 10.1017/S0031182018002184 30638174

[jane70106-bib-0003] Alerstam, T. , Hedenström, A. , & Åkesson, S. (2003). Long‐distance migration: Evolution and determinants. Oikos, 103(2), 247–260. 10.1034/j.1600-0706.2003.12559.x

[jane70106-bib-0004] Applegate, J. E. (1971). Spring relapse of Plasmodium relictum infections in an experimental field population of english sparrows (*Passer domesticus*). Journal of Wildlife Diseases, 7(1), 37–42. 10.7589/0090-3558-7.1.37 5138979

[jane70106-bib-0005] Arizaga, J. , Barba, E. , & Hernández, M. (2009). Do haemosporidians affect fuel deposition rate and fuel load in migratory blackcyps sylvia atricapilla? Ardeola, 56(1), 41–47.

[jane70106-bib-0006] Asghar, M. , Hasselquist, D. , & Bensch, S. (2011). Are chronic avian haemosporidian infections costly in wild birds? Journal of Avian Biology, 42(6), 530–537. 10.1111/j.1600-048X.2011.05281.x

[jane70106-bib-0007] Asghar, M. , Hasselquist, D. , Hansson, B. , Zehtindjiev, P. , Westerdahl, H. , & Bensch, S. (2015). Hidden costs of infection: Chronic malaria accelerates telomere degradation and senescence in wild birds. Science, 347(6220), 436–438. 10.1126/science.1261121 25613889

[jane70106-bib-0008] Atkinson, C. T. , Dusek, R. J. , & Lease, J. K. (2001). Serological responses and immunity to superinfection with avian malaria in experimentally‐infected Hawaii Amakihi. Journal of Wildlife Diseases, 37(1), 20–27. 10.7589/0090-3558-37.1.20 11272498

[jane70106-bib-0009] Atkinson, C. T. , Woods, K. L. , Dusek, R. J. , Sileo, L. S. , & Iko, W. M. (1995). Wildlife disease and conservation in Hawaii: Pathogenicity of avian malaria (*Plasmodium relictum*) in experimentally infected Iiwi (*Vestiaria coccinea*). Parasitology, 111(S1), S59–S69. 10.1017/S003118200007582X 8632925

[jane70106-bib-0010] Bairlain, F. , Jenni, L. , Kaiser, A. , Karlsson, L. , van Noordwijk, A. , Peach, W. , Pilastro, A. , Spina, F. , & Walinder, G. (1995). Manual of field methods. https://ifv‐vogelwarte.de/fileadmin/resources/Beringerzentrale/esf_manual.pdf

[jane70106-bib-0011] Bennett, G. , Caines, J. , & Bishop, M. (1988). Influence of blood parasites on the body‐mass of passeriform birds. Journal of Wildlife Diseases, 24(2), 339–343. 10.7589/0090-3558-24.2.339 3373640

[jane70106-bib-0012] Bensch, S. , Waldenström, J. , Jonzén, N. , Westerdahl, H. , Hansson, B. , Sejberg, D. , & Hasselquist, D. (2007). Temporal dynamics and diversity of avian malaria parasites in a single host species. Journal of Animal Ecology, 76(1), 112–122. 10.1111/j.1365-2656.2006.01176.x 17184359

[jane70106-bib-0013] Bichet, C. , Brischoux, F. , Ribout, C. , Parenteau, C. , Meillère, A. , & Angelier, F. (2020). Physiological and morphological correlates of blood parasite infection in urban and non‐urban house sparrow populations. PLoS One, 15(8), e0237170. 10.1371/journal.pone.0237170 32813710 PMC7437892

[jane70106-bib-0014] Binning, S. A. , Craft, M. E. , Zuk, M. , & Shaw, A. K. (2022). How to study parasites and host migration: A roadmap for empiricists. Biological Reviews, 97(3), 1161–1178. 10.1111/brv.12835 35094460

[jane70106-bib-0015] Binning, S. A. , Shaw, A. K. , & Roche, D. G. (2017). Parasites and host performance: Incorporating infection into our understanding of animal movement. Integrative and Comparative Biology, 57(2), 267–280. 10.1093/icb/icx024 28859405

[jane70106-bib-0016] Bürkner, P.‐C. (2017). Brms: An R package for Bayesian multilevel models using Stan. Journal of Statistical Software, 80, 1–28. 10.18637/jss.v080.i01

[jane70106-bib-0017] Carrier‐Belleau, C. , Pascal, L. , Tiegs, S. D. , Nozais, C. , & Archambault, P. (2023). Tipping point arises earlier under a multiple‐stressor scenario. Scientific Reports, 13(1), 16780. 10.1038/s41598-023-44012-x 37798389 PMC10555998

[jane70106-bib-0018] Cerini, F. , Childs, D. Z. , & Clements, C. F. (2023). A predictive timeline of wildlife population collapse. Nature Ecology & Evolution, 7(3), 320–331. 10.1038/s41559-023-01985-2 36702859

[jane70106-bib-0019] Chakarov, N. , & Blanco, G. (2021). Blood parasites in sympatric vultures: Role of nesting habits and effects on body condition. International Journal of Environmental Research and Public Health, 18(5), 2431. 10.3390/ijerph18052431 33801498 PMC7967578

[jane70106-bib-0020] Chen, F. , Jiang, F. , Ma, J. , Alghamdi, M. A. , Zhu, Y. , & Yong, J. W. H. (2024). Intersecting planetary health: Exploring the impacts of environmental stressors on wildlife and human health. Ecotoxicology and Environmental Safety, 283, 116848. 10.1016/j.ecoenv.2024.116848 39116691

[jane70106-bib-0021] Chrétien, E. , De Bonville, J. , Guitard, J. , Binning, S. A. , Melis, É. , Kack, A. , Côté, A. , Gradito, M. , Papillon, A. , Thelamon, V. , Levet, M. , & Barou‐Dagues, M. (2023). Few studies of wild animal performance account for parasite infections: A systematic review. Journal of Animal Ecology, 92(4), 794–806. 10.1111/1365-2656.13864 36480357

[jane70106-bib-0022] Clark, G. A. (1979). Body weights of birds: A review. The Condor, 81(2), 193. 10.2307/1367288

[jane70106-bib-0023] Clark, N. J. , Clegg, S. M. , & Lima, M. R. (2014). A review of global diversity in avian haemosporidians (Plasmodium and Haemoproteus: Haemosporida): New insights from molecular data. International Journal for Parasitology, 44(5), 329–338. 10.1016/j.ijpara.2014.01.004 24556563

[jane70106-bib-0024] Clements, C. F. , McCarthy, M. A. , & Blanchard, J. L. (2019). Early warning signals of recovery in complex systems. Nature Communications, 10(1), 1681. 10.1038/s41467-019-09684-y PMC6459826 30975997

[jane70106-bib-0025] Clements, C. F. , & Ozgul, A. (2016). Including trait‐based early warning signals helps predict population collapse. Nature Communications, 7(1), 10984. 10.1038/ncomms10984 PMC4820807 27009968

[jane70106-bib-0026] Cohen, J. (2013). Statistical power analysis for the behavioral sciences. Routledge. 10.4324/9780203771587

[jane70106-bib-0027] Coon, C. A. C. , Garcia‐Longoria, L. , Martin, L. B. , Magallanes, S. , de Lope, F. , & Marzal, A. (2016). Malaria infection negatively affects feather growth rate in the house sparrow *Passer domesticus* . Journal of Avian Biology, 47(6), 779–787. 10.1111/jav.00942

[jane70106-bib-0028] Coon, C. A. C. , & Martin, L. B. (2014). Patterns of haemosporidian prevalence along a range expansion in introduced Kenyan house sparrows *Passer domesticus* . Journal of Avian Biology, 45(1), 34–42. 10.1111/j.1600-048X.2013.00235.x

[jane70106-bib-0029] Cosgrove, C. L. , Wood, M. J. , Day, K. P. , & Sheldon, B. C. (2008). Seasonal variation in Plasmodium prevalence in a population of blue tits Cyanistes caeruleus. Journal of Animal Ecology, 77(3), 540–548. 10.1111/j.1365-2656.2008.01370.x 18312339

[jane70106-bib-0030] Costa, J. S. , Hahn, S. , Araújo, P. M. , Dhanjal‐Adams, K. L. , Rocha, A. D. , & Alves, J. A. (2021). Linking migratory performance to breeding phenology and productivity in an Afro‐Palearctic long‐distance migrant. Scientific Reports, 11(1), 23258. 10.1038/s41598-021-01734-0 34853345 PMC8636482

[jane70106-bib-0031] Culina, A. , Linton, D. M. , Pradel, R. , Bouwhuis, S. , & Macdonald, D. W. (2019). Live fast, don't die young: Survival–reproduction trade‐offs in long‐lived income breeders. Journal of Animal Ecology, 88(5), 746–756. 10.1111/1365-2656.12957 30737781 PMC6850603

[jane70106-bib-0032] Dadam, D. , Robinson, R. A. , Clements, A. , Peach, W. J. , Bennett, M. , Rowcliffe, J. M. , & Cunningham, A. A. (2019). Avian malaria‐mediated population decline of a widespread iconic bird species. Royal Society Open Science, 6(7), 182197. 10.1098/rsos.182197 31417708 PMC6689627

[jane70106-bib-0033] Daszak, P. , Cunningham, A. A. , & Hyatt, A. D. (2000). Emerging infectious diseases of wildlife—Threats to biodiversity and human health. Science, 287(5452), 443–449. 10.1126/science.287.5452.443 10642539

[jane70106-bib-0034] Davidar, P. , & Morton, E. (2006). Are multiple infections more severe for Purple Martins (*Progne subis*) than single infections? Auk, 123(1), 141–147. 10.1642/0004-8038(2006)123[0141:AMIMSF]2.0.CO;2

[jane70106-bib-0035] De Amaral, F. , Wilson, R. , Sonsthagen, S. , & Sehgal, R. (2023). Diversity, distribution, and methodological considerations of haemosporidian infections among Galliformes in Alaska. International Journal for Parasitology‐Parasites and Wildlife, 20, 122–132. 10.1016/j.ijppaw.2023.01.008 36798510 PMC9926109

[jane70106-bib-0036] De La Torre, G. M. , Freitas, F. F. , Fratoni, R. D. O. , Guaraldo, A. D. C. , Dutra, D. D. A. , Braga, M. , & Manica, L. T. (2020). Hemoparasites and their relation to body condition and plumage coloration of the White‐necked thrush (*Turdus albicollis*). Ethology Ecology & Evolution, 32(6), 509–526. 10.1080/03949370.2020.1769739

[jane70106-bib-0037] Dunn, J. , Goodman, S. , Benton, T. , & Hamer, K. (2013). Avian blood parasite infection during the non‐breeding season: An overlooked issue in declining populations? BMC Ecology, 13, 30. 10.1186/1472-6785-13-30 24011390 PMC3848531

[jane70106-bib-0038] Durlak, J. A. (2009). How to select, calculate, and interpret effect sizes. Journal of Pediatric Psychology, 34(9), 917–928. 10.1093/jpepsy/jsp004 19223279

[jane70106-bib-0039] Emmenegger, T. , Bauer, S. , Hahn, S. , Müller, S. B. , Spina, F. , & Jenni, L. (2018). Blood parasites prevalence of migrating passerines increases over the spring passage period. Journal of Zoology, 306(1), 23–27. 10.1111/jzo.12565

[jane70106-bib-0040] Emmenegger, T. , Bensch, S. , Hahn, S. , Kishkinev, D. , Procházka, P. , Zehtindjiev, P. , & Bauer, S. (2021). Effects of blood parasite infections on spatiotemporal migration patterns and activity budgets in a long‐distance migratory passerine. Ecology and Evolution, 11(2), 753–762. 10.1002/ece3.7030 33520163 PMC7820147

[jane70106-bib-0041] Ezenwa, V. , Archie, E. A. , Craft, M. E. , Hawley, D. M. , Martin, L. B. , Moore, J. , & White, L. (2016). Host behaviour–parasite feedback: An essential link between animal behaviour and disease ecology. Proceedings of the Royal Society B: Biological Sciences, 283(1828), 20153078. 10.1098/rspb.2015.3078 PMC4843650 27053751

[jane70106-bib-0042] Fecchio, A. , Clark, N. J. , Bell, J. A. , Skeen, H. R. , Lutz, H. L. , De La Torre, G. M. , Vaughan, J. A. , Tkach, V. V. , Schunck, F. , Ferreira, F. C. , Braga, É. M. , Lugarini, C. , Wamiti, W. , Dispoto, J. H. , Galen, S. C. , Kirchgatter, K. , Sagario, M. C. , Cueto, V. R. , González‐Acuña, D. , … Wells, K. (2021). Global drivers of avian haemosporidian infections vary across zoogeographical regions. Global Ecology and Biogeography, 30(12), 2393–2406. 10.1111/geb.13390

[jane70106-bib-0043] Figuerola, J. , Muñoz, E. , Gutiérrez, R. , & Ferrer, D. (1999). Blood parasites, leucocytes and plumage brightness in the Cirl Bunting, *Emberiza cirlus* . Functional Ecology, 13(5), 594–601. 10.1046/j.1365-2435.1999.00354.x

[jane70106-bib-0044] Gamelon, M. , Jenouvrier, S. , Lindner, M. , Sæther, B.‐E. , & Visser, M. E. (2023). Detecting climate signals cascading through levels of biological organization. Nature Climate Change, 13(9), 985–989. 10.1038/s41558-023-01760-y

[jane70106-bib-0045] Garvin, M. , Szell, C. , & Moore, F. (2006). Blood parasites of Nearctic‐Neotropical migrant passerine birds during spring trans‐gulf migration: Impact on host body condition. Journal of Parasitology, 92(5), 990–996. 10.1645/GE-758R.1 17152939

[jane70106-bib-0046] Gelman, A. , & Rubin, D. B. (1992). Inference from iterative simulation using multiple sequences. Statistical Science, 7(4), 457–472. 10.1214/ss/1177011136

[jane70106-bib-0047] Grabow, M. (2024). Subtle stressors—Strong responses. Consistent negative effects of avian blood parasites on phenotypic and demographic traits across songbirds [Dataset]. *Zenodo* . 10.5281/ZENODO.14420924 PMC12484424 40650477

[jane70106-bib-0048] Grabow, M. , Landgraf, C. , Niedballa, J. , Scholz, C. , Pufelski, J. , Nathan, R. , Toledo, S. , Jeltsch, F. , Blaum, N. , Radchuk, V. , Tiedemann, R. , & Kramer‐Schadt, S. (2025). Pathogen‐induced alterations in fine‐scale movement behaviour predict impaired reproductive success. Proceedings of the Royal Society B: Biological Sciences, 292(2044), 20250238. 10.1098/rspb.2025.0238 PMC11978449 40199355

[jane70106-bib-0049] Grabow, M. , Ullmann, W. , Landgraf, C. , Sollmann, R. , Scholz, C. , Nathan, R. , Toledo, S. , Lühken, R. , Fickel, J. , Jeltsch, F. , Blaum, N. , Radchuk, V. , Tiedemann, R. , & Kramer‐Schadt, S. (2024). Sick without signs. Subclinical infections reduce local movements, alter habitat selection, and cause demographic shifts. Communications Biology, 7(1), 1426. 10.1038/s42003-024-07114-4 39487334 PMC11530534

[jane70106-bib-0050] Grenfell, B. T. , & Dobson, A. P. (Eds.). (2008). Ecology of infectious diseases in natural populations (Digitally printed version). Cambridge University Press.

[jane70106-bib-0051] Guglielmo, C. G. (2018). Obese super athletes: Fat‐fueled migration in birds and bats. Journal of Experimental Biology, 221(Suppl_1), jeb165753. 10.1242/jeb.165753 29514885

[jane70106-bib-0052] Hahn, S. , Bauer, S. , Dimitrov, D. , Emmenegger, T. , Ivanova, K. , Zehtindjiev, P. , & Buttemer, W. A. (2018). Low intensity blood parasite infections do not reduce the aerobic performance of migratory birds. Proceedings. Biological sciences, 285(1871), 20172307. 10.1098/rspb.2017.2307 29386365 PMC5805937

[jane70106-bib-0053] Hall, R. J. , Altizer, S. , Peacock, S. J. , & Shaw, A. K. (2022). Animal migration and infection dynamics: Recent advances and future frontiers. In V. Ezenwa , S. M. Altizer , & R. Hall (Eds.), Animal behavior and parasitism (1st ed., pp. 111–132). University Press Oxford. 10.1093/oso/9780192895561.003.0007

[jane70106-bib-0054] Hall, R. J. , Brown, L. M. , & Altizer, S. (2016). Modeling vector‐borne disease risk in migratory animals under climate change. Integrative and Comparative Biology, 56(2), 353–364. 10.1093/icb/icw049 27252225

[jane70106-bib-0055] Hamilton, W. D. , & Zuk, M. (1982). Heritable true fitness and bright birds: A role for parasites? Science, 218(4570), 384–387. 10.1126/science.7123238 7123238

[jane70106-bib-0056] Hammers, M. , Komdeur, J. , Kingma, S. A. , Hutchings, K. , Fairfield, E. A. , Gilroy, D. L. , & Richardson, D. S. (2016). Age‐specific haemosporidian infection dynamics and survival in Seychelles warblers. Scientific Reports, 6(1), 29720. 10.1038/srep29720 27431430 PMC4949462

[jane70106-bib-0057] Hatchwell, B. , Wood, M. , Anwar, M. , Chamberlain, D. , & Perrins, C. (2001). The haematozoan parasites of Common Blackbirds *Turdus merula*: Associations with host condition. Ibis, 143(3), 420–426. 10.1111/ibi.2001.143.4.420

[jane70106-bib-0058] Hawley, D. M. , & Altizer, S. M. (2011). Disease ecology meets ecological immunology: Understanding the links between organismal immunity and infection dynamics in natural populations. Functional Ecology, 25(1), 48–60. 10.1111/j.1365-2435.2010.01753.x

[jane70106-bib-0059] Hedges, L. V. (1981). Distribution theory for glass's estimator of effect size and related estimators. Journal of Educational Statistics, 6(2), 107. 10.2307/1164588

[jane70106-bib-0060] Hegemann, A. , Alcalde Abril, P. , Muheim, R. , Sjöberg, S. , Alerstam, T. , Nilsson, J. Å. , & Hasselquist, D. (2018). Immune function and blood parasite infections impact stopover ecology in passerine birds. Oecologia, 188(4), 1011–1024. 10.1007/s00442-018-4291-3 30386941 PMC6244813

[jane70106-bib-0061] Hellgren, O. , Waldenström, J. , & Bensch, S. (2004). A new PCR assay for simultaneous studies of Leucocytozoon, Plasmodium, and Haemoproteus from avian blood. The Journal of Parasitology, 90(4), 797–802. 10.1645/GE-184R1 15357072

[jane70106-bib-0062] Hernández, M. , Rojo, M. , Campos, F. , Gutiérrez‐Corchero, F. , & Moreno‐Rueda, G. (2020). Haemosporidian prevalence in southern Grey Shrike Lanius meridionalis nestlings in the Iberian Peninsula: Lower prevalence than previously reported. Bird Study, 67(3), 398–401. 10.1080/00063657.2020.1864286

[jane70106-bib-0063] Hernandez‐Colina, A. , Gonzalez‐Olvera, M. , Eckley, L. , Lopez, J. , & Baylis, M. (2021). Avian malaria affecting penguins in zoological gardens, aquariums and wildlife parks in the UK. Veterinary Record, 189(9), e511. 10.1002/vetr.511 34019706

[jane70106-bib-0064] Hicks, O. , Burthe, S. J. , Daunt, F. , Newell, M. , Butler, A. , Ito, M. , Sato, K. , & Green, J. A. (2018). The energetic cost of parasitism in a wild population. Proceedings of the Royal Society B: Biological Sciences, 285(1879), 20180489. 10.1098/rspb.2018.0489 PMC5998108 29848646

[jane70106-bib-0065] Hughes, A. C. , Orr, M. C. , Ma, K. , Costello, M. J. , Waller, J. , Provoost, P. , Yang, Q. , Zhu, C. , & Qiao, H. (2021). Sampling biases shape our view of the natural world. Ecography, 44(9), 1259–1269. 10.1111/ecog.05926

[jane70106-bib-0066] Ings, K. , & Denk, D. (2022). Avian malaria in penguins: Diagnostics and future direction in the context of climate change. Animals, 12(5), 600. 10.3390/ani12050600 35268169 PMC8909384

[jane70106-bib-0067] Jiménez‐Gallardo, L. , López‐Arrabé, J. , Pérez‐Tris, J. , & Remacha, C. (2025). Young male blackcaps with blood parasite coinfections cope with oxidative stress favouring anthocyanin‐rich food during migratory fattening. Journal of Avian Biology, 2025(2), e03214. 10.1111/jav.03214

[jane70106-bib-0068] Kaiser, A. (1993). A new multi‐category classification of subcutaneous fat deposits of songbirds. Journal of Field Ornithology, 64, 246–255. https://www.semanticscholar.org/paper/A‐new‐multi‐category‐classification‐of‐subcutaneous‐Kaiser/e841610838a734b3d1f7b0d1a38d5cd67768e012

[jane70106-bib-0069] Kelly, T. R. , Rubin, B. D. , MacDougall‐Shackleton, S. A. , & MacDougall‐Shackleton, E. A. (2020). Experimental malaria infection affects songbirds' nocturnal migratory activity. Physiological and Biochemical Zoology, 93(2), 97–110. 10.1086/707495 32013740

[jane70106-bib-0070] Kilpatrick, A. M. , LaPointe, D. A. , Atkinson, C. T. , Woodworth, B. L. , Lease, J. K. , Reiter, M. E. , & Gross, K. (2006). Effects of chronic avian malaria (*Plasmodium relictum*) infection on reproductive success of Hawaii Amakihi (*Hemignathus virens*). The Auk, 123(3), 764–774. 10.1093/auk/123.3.764

[jane70106-bib-0071] Koltz, A. M. , Civitello, D. J. , Becker, D. J. , Deem, S. L. , Classen, A. T. , Barton, B. , Brenn‐White, M. , Johnson, Z. E. , Kutz, S. , Malishev, M. , Preston, D. L. , Vannatta, J. T. , Penczykowski, R. M. , & Ezenwa, V. O. (2022). Sublethal effects of parasitism on ruminants can have cascading consequences for ecosystems. Proceedings of the National Academy of Sciences of the United States of America, 119(20), e2117381119. 10.1073/pnas.2117381119 35533278 PMC9171767

[jane70106-bib-0072] Koricheva, J. , Gurevitch, J. , & Mengersen, K. (Eds.). (2013). Handbook of meta‐analysis in ecology and evolution. Princeton University Press.

[jane70106-bib-0073] Krause, J. S. , Pérez, J. H. , Meddle, S. L. , & Wingfield, J. C. (2017). Effects of short‐term fasting on stress physiology, body condition, and locomotor activity in wintering male white‐crowned sparrows. Physiology & Behavior, 177, 282–290. 10.1016/j.physbeh.2017.04.026 28472668

[jane70106-bib-0074] Kubacka, J. , Gerlée, A. , Foucher, J. , Korb, J. , & Podmokla, E. (2019). Correlates of blood parasitism in a threatened marshland passerine: Infection by kinetoplastids of the genus Trypanosoma is related to landscape metrics of habitat edge. Parasitology, 146(8), 1036–1046. 10.1017/S0031182019000350 31064439

[jane70106-bib-0075] López, G. , Muñoz, J. , Soriguer, R. , & Figuerola, J. (2013). Increased Endoparasite infection in late‐arriving individuals of a Trans‐Saharan passerine migrant bird. PLoS One, 8(4), e61236. 10.1371/journal.pone.0061236 23620731 PMC3631203

[jane70106-bib-0076] Lüdecke, D. (2018). *esc*: *Effect size computation for meta analysis* (Version 0.4.1) [Computer software]. *Zenodo*. 10.5281/ZENODO.1249218

[jane70106-bib-0077] Magallanes, S. , García‐Longoria, L. , López‐Calderón, C. , Reviriego, M. , de Lope, F. , Moller, A. , & Marzal, A. (2017). Uropygial gland volume and malaria infection are related to survival in migratory house martins. Journal of Avian Biology, 48(11), 1355–1359. 10.1111/jav.01514

[jane70106-bib-0078] Marzal, A. , Asghar, M. , Rodríguez, L. , Reviriego, M. , Hermosell, I. , Balbontín, J. , Garcia‐Longoria, L. , de Lope, F. , & Bensch, S. (2013). Co‐infections by malaria parasites decrease feather growth but not feather quality in house martin. Journal of Avian Biology, 44(5), 437–444. 10.1111/j.1600-048X.2013.00178.x

[jane70106-bib-0079] Marzal, A. , Bensch, S. , Reviriego, M. , Balbontin, J. , & De Lope, F. (2008). Effects of malaria double infection in birds: One plus one is not two. Journal of Evolutionary Biology, 21(4), 979–987. 10.1111/j.1420-9101.2008.01545.x 18462316

[jane70106-bib-0080] Marzal, A. , De Lope, F. , Navarro, C. , & Møller, A. P. (2005). Malarial parasites decrease reproductive success: An experimental study in a passerine bird. Oecologia, 142(4), 541–545. 10.1007/s00442-004-1757-2 15688214

[jane70106-bib-0081] Marzal, A. , Reviriego, M. , Hermosell, I. G. , Balbontín, J. , Bensch, S. , Relinque, C. , Rodríguez, L. , Garcia‐Longoria, L. , & de Lope, F. (2013). Malaria infection and feather growth rate predict reproductive success in house martins. Oecologia, 171(4), 853–861. 10.1007/s00442-012-2444-3 22961369

[jane70106-bib-0082] McElroy, E. J. , & de Buron, I. (2014). Host performance as a target of manipulation by parasites: A meta‐analysis. Journal of Parasitology, 100(4), 399–410. 10.1645/13-488.1 24766282

[jane70106-bib-0083] Merilä, J. , & Andersson, M. (1999). Reproductive effort and success are related to haematozoan infections in blue tits. Ecoscience, 6(3), 421–428. 10.1080/11956860.1999.11682542

[jane70106-bib-0084] Merino, S. , Moreno, J. , Sanz, J. , & Arriero, E. (2000). Are avian blood parasites pathogenic in the wild?: A medication experiment in blue tits (*Parus caeruleus*). Proceedings of the Royal Society B: Biological Sciences, 267(1461), 2507–2510. 10.1098/rspb.2000.1312 PMC1690848 11197126

[jane70106-bib-0085] Moller, A. , De Lope, F. , & Saino, N. (2004). Parasitism, immunity, and arrival date in a migratory bird, the barn swallow. Ecology, 85(1), 206–219. 10.1890/02-0451

[jane70106-bib-0086] Mora‐Rubio, C. , Garcia‐Longoria, L. , Ferraguti, M. , Magallanes, S. , Cruz, J. , de Lope, F. , & Marzal, A. (2024). The impact of avian Haemosporidian infection on feather quality and feather growth rate of migratory passerines. Animals, 14(12), 1772. 10.3390/ani14121772 38929391 PMC11200494

[jane70106-bib-0087] Mukhin, A. , Palinauskas, V. , Platonova, E. , Kobylkov, D. , Vakoliuk, I. , & Valkiūnas, G. (2016). The strategy to survive primary malaria infection: An experimental study on behavioural changes in parasitized birds. PLoS One, 11(7), e0159216. 10.1371/journal.pone.0159216 27434058 PMC4951008

[jane70106-bib-0088] Muriel, J. , Garcia‐Longoria, L. , Magallanes, S. , Ortiz, J. , & Marzal, A. (2023). Avian malaria, haematocrit, and body condition in invasive wetland passerines settled in southwestern Spain. Avian Research, 14, 100081. 10.1016/j.avrs.2023.100081

[jane70106-bib-0089] Nakagawa, S. , Lagisz, M. , Jennions, M. D. , Koricheva, J. , Noble, D. W. A. , Parker, T. H. , Sánchez‐Tójar, A. , Yang, Y. , & O'Dea, R. E. (2022). Methods for testing publication bias in ecological and evolutionary meta‐analyses. Methods in Ecology and Evolution, 13(1), 4–21. 10.1111/2041-210X.13724

[jane70106-bib-0090] Norte, A. , Araújo, P. , Sampaio, H. , Sousa, J. , & Ramos, J. (2009). Haematozoa infections in a Great Tit Parus major population in Central Portugal: Relationships with breeding effort and health. Ibis, 151(4), 677–688. 10.1111/j.1474-919X.2009.00960.x

[jane70106-bib-0091] Oppliger, A. , Christe, P. , & Richner, H. (1997). Clutch size and malarial parasites in female great tits. Behavioral Ecology, 8(2), 148–152. 10.1093/beheco/8.2.148

[jane70106-bib-0092] Ots, I. , & Horak, P. (1996). Great tits Parus major trade health for reproduction. Proceedings of the Royal Society B: Biological Sciences, 263(1376), 1443–1447. 10.1098/rspb.1996.0210 8952087

[jane70106-bib-0093] Page, M. J. , McKenzie, J. E. , Bossuyt, P. M. , Boutron, I. , Hoffmann, T. C. , Mulrow, C. D. , Shamseer, L. , Tetzlaff, J. M. , Akl, E. A. , Brennan, S. E. , Chou, R. , Glanville, J. , Grimshaw, J. M. , Hróbjartsson, A. , Lalu, M. M. , Li, T. , Loder, E. W. , Mayo‐Wilson, E. , McDonald, S. , … Moher, D. (2021). The PRISMA 2020 statement: An updated guideline for reporting systematic reviews. BMJ (Clinical Research Ed.), 372, n71. 10.1136/bmj.n71 PMC8005924 33782057

[jane70106-bib-0094] Palinauskas, V. , Valkiūnas, G. , Bolshakov, C. V. , & Bensch, S. (2008). Plasmodium relictum (lineage P‐SGS1): Effects on experimentally infected passerine birds. Experimental Parasitology, 120(4), 372–380. 10.1016/j.exppara.2008.09.001 18809402

[jane70106-bib-0095] Pelletier, F. , Clutton‐Brock, T. , Pemberton, J. , Tuljapurkar, S. , & Coulson, T. (2007). The evolutionary demography of ecological change: Linking trait variation and population growth. Science, 315(5818), 1571–1574. 10.1126/science.1139024 17363672

[jane70106-bib-0096] Piersma, T. , & van der Velde, M. (2012). Dutch house martins *Delichon urbicum* gain blood parasite infections over their lifetime, but do not seem to suffer. Journal of Ornithology, 153(3), 907–912. 10.1007/s10336-012-0826-2

[jane70106-bib-0097] Pigeault, R. , Cozzarolo, C. , Choquet, R. , Strehler, M. , Jenkins, T. , Delhaye, J. , Bovet, L. , Wassef, J. , Glaizot, O. , & Christe, P. (2018). Haemosporidian infection and co‐infection affect host survival and reproduction in wild populations of great tits. International Journal for Parasitology, 48(14), 1079–1087. 10.1016/j.ijpara.2018.06.007 30391229

[jane70106-bib-0098] Poblete, Y. , Cuevas, É. , Botero‐Delgadillo, E. , Espindola‐Hernández, P. , Quirici, V. , & Vásquez, R. (2024). Risk‐taking behavior relates to Leucocytozoon spp. infection in a sub‐Antarctic rainforest bird. Acta Ethologica, 27(2), 113–123. 10.1007/s10211-024-00437-9

[jane70106-bib-0099] Podmokla, E. , Dubiec, A. , Drobniak, S. , Arct, A. , Gustafsson, L. , & Cichon, M. (2014). Avian malaria is associated with increased reproductive investment in the blue tit. Journal of Avian Biology, 45(3), 219–224. 10.1111/j.1600-048X.2013.00284.x

[jane70106-bib-0100] Podmokla, E. , Dubiec, A. , Drobniak, S. , Sudyka, J. , Krupski, A. , Arct, A. , Gustafsson, L. , & Cichon, M. (2017). Effect of haemosporidian infections on host survival and recapture rate in the blue tit. Journal of Avian Biology, 48(6), 796–803. 10.1111/jav.01108

[jane70106-bib-0101] Poulin, R. (2011). Evolutionary ecology of parasites (2nd ed.). Princeton University Press. 10.1515/9781400840809

[jane70106-bib-0102] R Core Team . (2024). R: A language and environment for statistical computing. R Foundation for Statistical Computing. https://www.R‐project.org/

[jane70106-bib-0103] Rätti, O. , Dufva, R. , & Alatalo, R. V. (1993). Blood parasites and male fitness in the pied flycatcher. Oecologia, 96(3), 410–414. 10.1007/BF00317512 28313657

[jane70106-bib-0104] Risely, A. , Klaassen, M. , & Hoye, B. J. (2018). Migratory animals feel the cost of getting sick: A meta‐analysis across species. Journal of Animal Ecology, 87(1), 301–314. 10.1111/1365-2656.12766 28994103

[jane70106-bib-0105] Romano, A. , Nodari, R. , Bandi, C. , Caprioli, M. , Costanzo, A. , Ambrosini, R. , Rubolini, D. , Parolini, M. , Epis, S. , & Saino, N. (2019). Haemosporidian parasites depress breeding success and plumage coloration in female barn swallows Hirundo rustica. Journal of Avian Biology, 50(2), jav.01889. 10.1111/jav.01889

[jane70106-bib-0106] Sanz, J. J. , Arriero, E. , Moreno, J. , & Merino, S. (2001). Interactions between hemoparasite status and female age in the primary reproductive output of pied flycatchers. Oecologia, 126(3), 339–344. 10.1007/s004420000530 28547446

[jane70106-bib-0107] Scheffer, M. , Bascompte, J. , Brock, W. A. , Brovkin, V. , Carpenter, S. R. , Dakos, V. , Held, H. , van Nes, E. H. , Rietkerk, M. , & Sugihara, G. (2009). Early‐warning signals for critical transitions. Nature, 461(7260), 53–59. 10.1038/nature08227 19727193

[jane70106-bib-0108] Schoenle, L. A. , Kernbach, M. , Haussmann, M. F. , Bonier, F. , & Moore, I. T. (2017). An experimental test of the physiological consequences of avian malaria infection. Journal of Animal Ecology, 86(6), 1483–1496. 10.1111/1365-2656.12753 28884826

[jane70106-bib-0109] Schoepf, I. , Olson, S. , Moore, I. T. , & Bonier, F. (2022). Experimental reduction of haemosporidian infection affects maternal reproductive investment, parental behaviour and offspring condition. Proceedings of the Royal Society B: Biological Sciences, 289(1987), 20221978. 10.1098/rspb.2022.1978 PMC9709520 36448284

[jane70106-bib-0110] Schrader, M. S. , Walters, E. L. , James, F. C. , & Greiner, E. C. (2003). Seasonal prevalence of a haematozoan parasite of Red‐bellied Woodpeckers (*Melanerpes carolinus*) and its association with host condition and overwinter survival. Auk, 120(1), 130–137. 10.2307/4090147

[jane70106-bib-0111] Schumm, Y. , Lederer‐Ponzer, N. , Masello, J. , & Quillfeldt, P. (2024). High prevalence of haemosporidian parasites in Eurasian jays. Parasitology Research, 123(4), 182. 10.1007/s00436-024-08170-9 38622257 PMC11018679

[jane70106-bib-0112] Shanebeck, K. M. , Besson, A. A. , Lagrue, C. , & Green, S. J. (2022). The energetic costs of sub‐lethal helminth parasites in mammals: A meta‐analysis. Biological Reviews, 97(5), 1886–1907. 10.1111/brv.12867 35678252

[jane70106-bib-0113] Sheldon, B. C. , & Verhulst, S. (1996). Ecological immunology: Costly parasite defences and trade‐offs in evolutionary ecology. Trends in Ecology & Evolution, 11(8), 317–321. 10.1016/0169-5347(96)10039-2 21237861

[jane70106-bib-0114] Shurulinkov, P. , Chakarov, N. , & Daskalova, G. (2012). Blood parasites, body condition, and wing length in two subspecies of yellow wagtail (*Motacilla flava*) during migration. Parasitology Research, 110(5), 2043–2051. 10.1007/s00436-011-2733-5 22278726

[jane70106-bib-0115] Smith, V. H. , Jones, T. P., II , & Smith, M. S. (2005). Host nutrition and infectious disease: An ecological view. Frontiers in Ecology and the Environment, 3(5), 268–274. 10.1890/1540-9295(2005)003[0268:HNAIDA]2.0.CO;2 PMC2762229 19838319

[jane70106-bib-0116] Stearns, S. C. (1992). The evolution of life histories. Oxford University Press. 10.1046/j.1420-9101.1993.6020304.x

[jane70106-bib-0117] Valkiunas, G. (2004). Avian malaria parasites and other haemosporidia. CRC Press.

[jane70106-bib-0118] Van Hemert, C. , Meixell, B. W. , Smith, M. M. , & Handel, C. M. (2019). Prevalence and diversity of avian blood parasites in a resident northern passerine. Parasites & Vectors, 12(1), 1–16. 10.1186/s13071-019-3545-1 31182151 PMC6558893

[jane70106-bib-0119] van Riper, C., III , van Riper, S. G. , Goff, M. L. , & Laird, M. (1986). The Epizootiology and ecological significance of malaria in Hawaiian land birds. Ecological Monographs, 56(4), 327–344. 10.2307/1942550

[jane70106-bib-0120] Vehtari, A. , Gelman, A. , & Gabry, J. (2017). Practical Bayesian model evaluation using leave‐one‐out cross‐validation and WAIC. Statistics and Computing, 27(5), 1413–1432. 10.1007/s11222-016-9696-4

[jane70106-bib-0121] Velando, A. , Drummond, H. , & Torres, R. (2006). Senescent birds redouble reproductive effort when ill: Confirmation of the terminal investment hypothesis. Proceedings of the Royal Society B: Biological Sciences, 273(1593), 1443–1448. 10.1098/rspb.2006.3480 PMC1560321 16777735

[jane70106-bib-0122] Volker, M. A. (2006). Reporting effect size estimates in school psychology research. Psychology in the Schools, 43(6), 653–672. 10.1002/pits.20176

[jane70106-bib-0123] Votypka, J. , Simek, J. , & Tryjanowski, P. (2003). Blood parasites, reproduction and sexual selection in the red‐backed shrike (*Lanius collurio*). Annales Zoologici Fennici, 40(5), 431–439.

[jane70106-bib-0124] Weatherhead, P. J. , & Bennett, G. F. (1992). Ecology of parasitism of Brown‐headed cowbirds by haematozoa. Canadian Journal of Zoology, 70(1), 1–7. 10.1139/z92-001

[jane70106-bib-0125] Williams, G. C. (1966). Natural selection, the costs of reproduction, and a refinement of Lack's principle. The American Naturalist, 100(916), 687–690. 10.1086/282461

[jane70106-bib-0126] Wobeser, G. A. (2006). Essentials of disease in wild animals (1st ed.). Blackwell.

[jane70106-bib-0127] Yorinks, N. , & Atkinson, C. T. (2000). Effects of malaria on activity budgets of experimentally infected juvenile Apapane (*Himatione sanguinea*). Auk, 117(3), 731–738. 10.2307/4089597

[jane70106-bib-0128] Yusupova, D. A. , Schumm, Y. R. , Sokolov, A. A. , & Quillfeldt, P. (2023). Haemosporidian blood parasites of passerine birds in north‐western Siberia. Polar Biology, 46(6), 497–511. 10.1007/s00300-023-03130-y

[jane70106-bib-0129] Zylberberg, M. , Derryberry, E. P. , Breuner, C. W. , Macdougall‐Shackleton, E. A. , Cornelius, J. M. , & Hahn, T. P. (2015). Haemoproteus infected birds have increased lifetime reproductive success. Parasitology, 142(8), 1033–1043. 10.1017/S0031182015000256 25800822

