# Supplementary material for: Subtle stressors—Strong responses. Consistent negative effects of avian blood parasites on phenotypic and demographic traits across songbirds
Source: J Anim Ecol. 2025 Jul 12;94(10):1908–21. doi: 10.1111/1365-2656.70106 (PMC12484424; doi:10.1111/1365-2656.70106)
Supplement: Supplementary file 1 — Figure S1. Conceptual framework illustrating the relationships between phenotypic and demographic traits, and their feedback to fitness. Table S2. Overview of phenotypic and demographic trait categories and the detailed traits that were included in each category. Sample size (n) describes number of effect sizes calculated for each trait category. Table S4. Model estimates from the original manuscript compared to estimates without the Hawaiian study system, which is known to differ from many other study systems due to the lack of evolutionary history between host and parasite. Figure S3. Identification of influential studies using Pareto k. Dashed orange line (k = 0.7) indicates influential studies, dashed red line (k = 1) indicates highly influential studies. Figure S5. Funnel plot for host condition. Each point represents one effect size, coloured by study. Figure S6. Funnel plot for host survival. Each point represents one effect size, coloured by study. Figure S7. Funnel plot for host reproduction. Each point represents one effect size, coloured by study. Figure S8. Funnel plot for phenology. Each point represents one effect size, coloured by study. Figure S9. Relationship between inverse effective sample size and effect sizes. Each point represents an individual study, with colours differentiating study categories. Blue line and shaded are represent predicted effect size and 95% CI. The symmetrical horizontal distribution indicates minimal bias regarding study size. Figure S10. Relationship between study year and effect sizes. Each point represents an individual study, with colours differentiating study categories. Blue line and shaded are represent predicted effect size and 95% CI. The symmetrical horizontal distribution indicates minimal bias regarding study year. [file JANE-94-1908-s001.docx]

Supplementary material for

**Subtle stressors – strong responses. Consistent negative effects of avian blood parasites on phenotypic and demographic traits across songbirds**

# S1 Conceptual framework
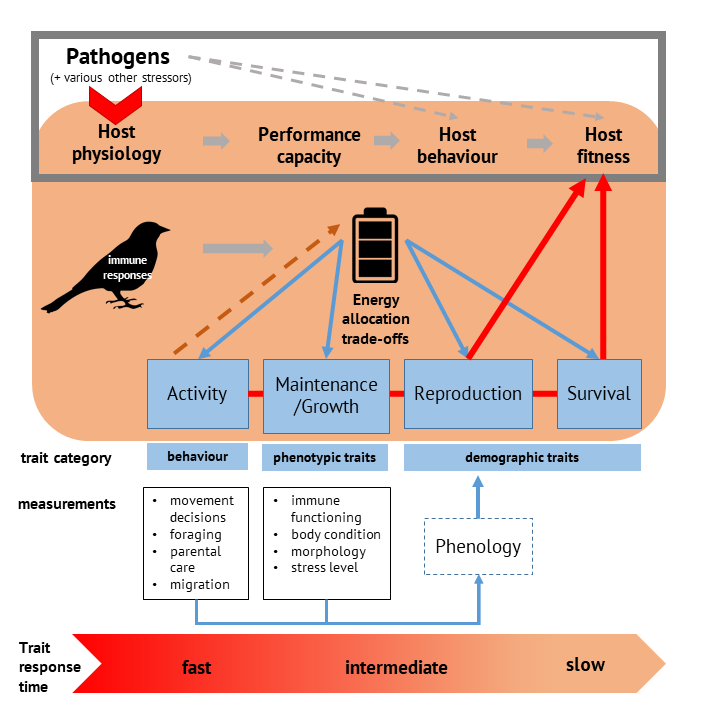


Figure S1: Conceptual framework illustrating the relationships between phenotypic and demographic traits, and their feedback to fitness. Pathogens disrupt physiological processes of hosts, altering performance capacity, behaviour, and eventually fitness (grey box, adapted from McElroy & Buron, 2014). This concept is closely linked to Life-history theory (orange box): Individuals allocate resources among competing life-history demands, e.g. investing in reproduction at the cost of reduced immune defence (trade-offs). In this context, activity (behaviour) plays a pivotal role by linking energy expenditure and gain (e.g., through foraging or migrating to resource-rich habitats). Measurable traits, i.e. behavioural traits, phenotypic traits, and demographic traits differ in their response times: immune responses occur immediately, movement behaviour adjusts rapidly, and demographic traits such as reduced reproduction, respond more slowly. In this context, measurements of one type of phenotypic traits, phenology, play a unique role, influencing demographic traits while being shaped by behavioural and other phenotypic traits (e.g., migration behaviour depends on energetic reserves, ultimately altering departure timing). Flexible traits like behaviour have the potential to cascade through biological levels, with impairments in individual behaviour affecting phenotypic traits, and eventually demographic traits that determine fitness (red arrow).

# S2 Trait categories

We categorised trait responses into four trait categories: (1) host condition (here defined broadly, incorporating morphological traits such as body condition, growth and physiological traits such as oxidative stress levels (see Supplementary Table S1 and Discussion S1), (2) phenology (e.g. timing of breeding or migration), (3) reproduction (e.g. fledging success), and (4) survival. A key challenge in this meta-analysis was to balance biological specificity and statistical power. While finer categorisation could capture mechanistic differences among trait categories, it would reduce statistical power and blur broader trends. For example, we categorized various phenotypic traits into ‘condition’ to maintain analytical feasibility while still capturing biologically meaningful effects of physiological, morphology, and performance.

Such classification is supported by the fact that all included traits reflect a host’s physiological state and resource allocation. Although traits like tarsus length and body mass could also indicate structural differences, they remain relevant in the context of infection, with numerous studies reporting reduced growth and developmental constraints in infected individuals. Similarly ‘struggle in hand’ and ‘access to quality habitat’ were included, given that physiological stress may directly influence behaviour and habitat selection, even if these traits also have personality-driven variation. Therefore, while alternative plausible explanations exist for our chosen trait categories, a unifying biological mechanism unites them: infection induces alterations in energy balance due to various causes, altering resource acquisition, and overall host health.

Finally, we acknowledge that our trait categorization inevitably introduces heterogeneity within trait categories, however, further breaking down categories would reduce statistical power and robustness, as many traits are sparsely represented in the literature (Table S1). By summarising them as ‘condition’, we provide a biologically meaningful and statistically robust framework to assess the generalized effects of infection across various host responses.

Table S2: Overview of phenotypic and demographic trait categories and the detailed traits that were included in each category. Sample size (n) describes number of effect sizes calculated for each trait category.

| Trait category | n | Detailed phenotypic traits included |
| --- | --- | --- |
| Condition | 117 | body mass (n=46) |
|  |  | Body mass loss (n=1) |
|  |  | body condition (n=36) |
|  |  | fat score (n=9) |
|  |  | stress level (n=4) |
|  |  | struggle in hand (n=3) |
|  |  | feather growth rate (n=3) |
|  |  | feather quality (n=2) |
|  |  | growth bar width (n=2) |
|  |  | growth bar number (n=1) |
|  |  | rectrix feather length (n=1) |
|  |  | tail asymmetry (n=1) |
|  |  | tail patch size (n=1) |
|  |  | bill colour (n=2) |
|  |  | tarsus length (n=1) |
|  |  | access to quality habitat (n=1) |
| Phenology | 16 | egg laying date (n=6) |
|  |  | breeding timing (n=2) |
|  |  | spring arrival during migration (n=6) |
|  |  | stopover duration during migration (n=2) |
| Reproduction | 31 | clutch size (n=12) |
|  |  | brood size (n=7) |
|  |  | hatching success (n=2) |
|  |  | fledging success (n=3) |
|  |  | egg weight (n=2) |
|  |  | clutch volume (n=1) |
|  |  | parental effort (n=4) |
| survival | 8 | Survival (n=8) |

# Influential studies in meta-analysis


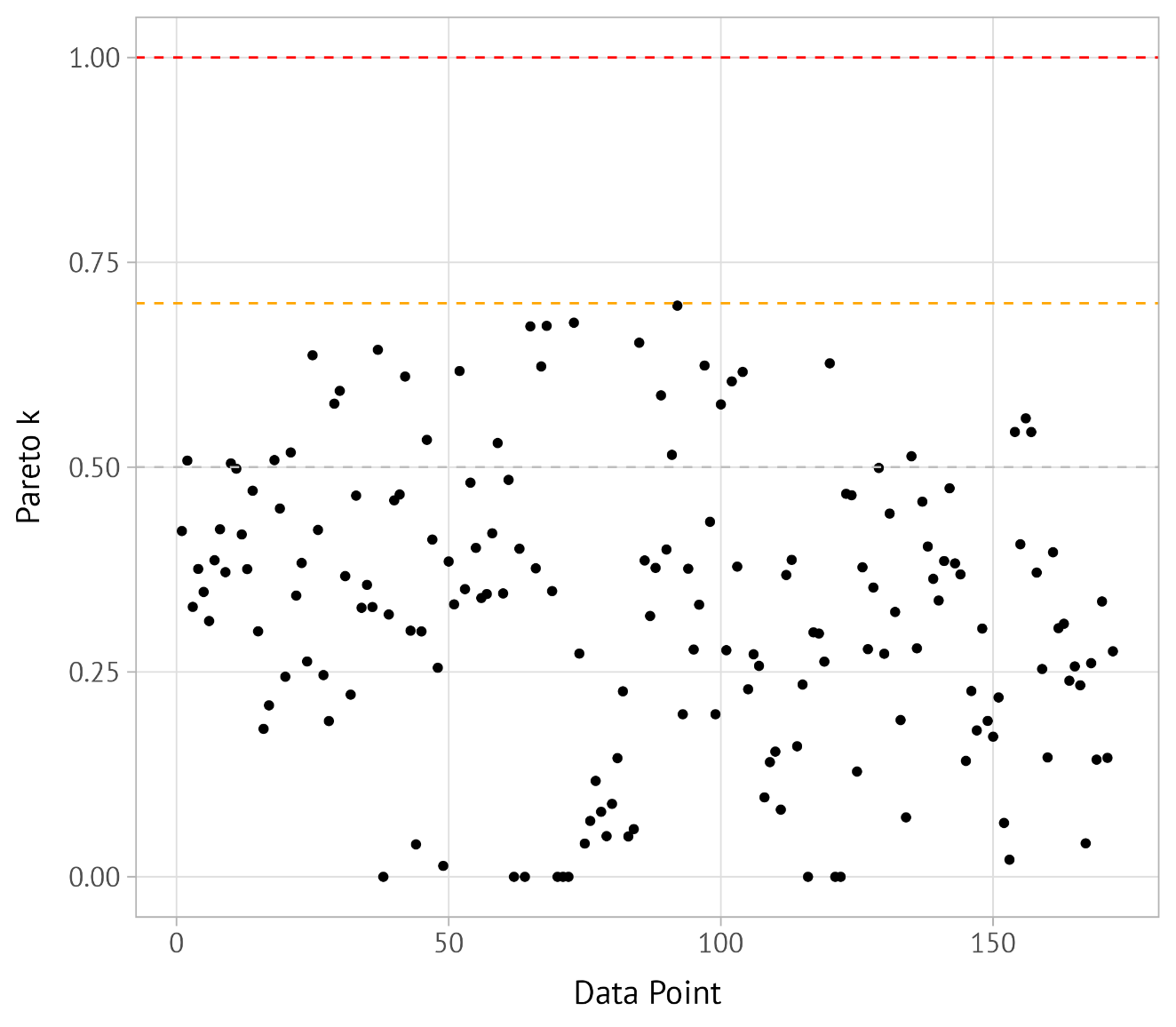


Figure S3: Identification of influential studies using Pareto k. Dashed orange line (k=0.7) indicates influential studies, dashed red line (k=1) indicates highly influential studies.

Table S4: Model estimates from the original manuscript compared to estimates without the Hawaiian study system, which is known to differ from many other study systems due to the lack of evolutionary history between host and parasite.

| Trait category | Estimate (original MS) | Estimate (without Hawaii) |
| --- | --- | --- |
| Condition | -0.15 [-0.30, 0.00] | -0.16 [-0.31, 0.00] |
| Phenology | 0.25 [0.06, 0.44] | 0.24 [0.05, 0.43] |
| Reproduction | -0.25 [-0.42, -0.08] | -0.27 [-0.45, -0.10] |
| Survival | -0.40 [-0.61, -0.20] | -0.41 [-0.62, -0.21] |

# Funnel plots

## Condition


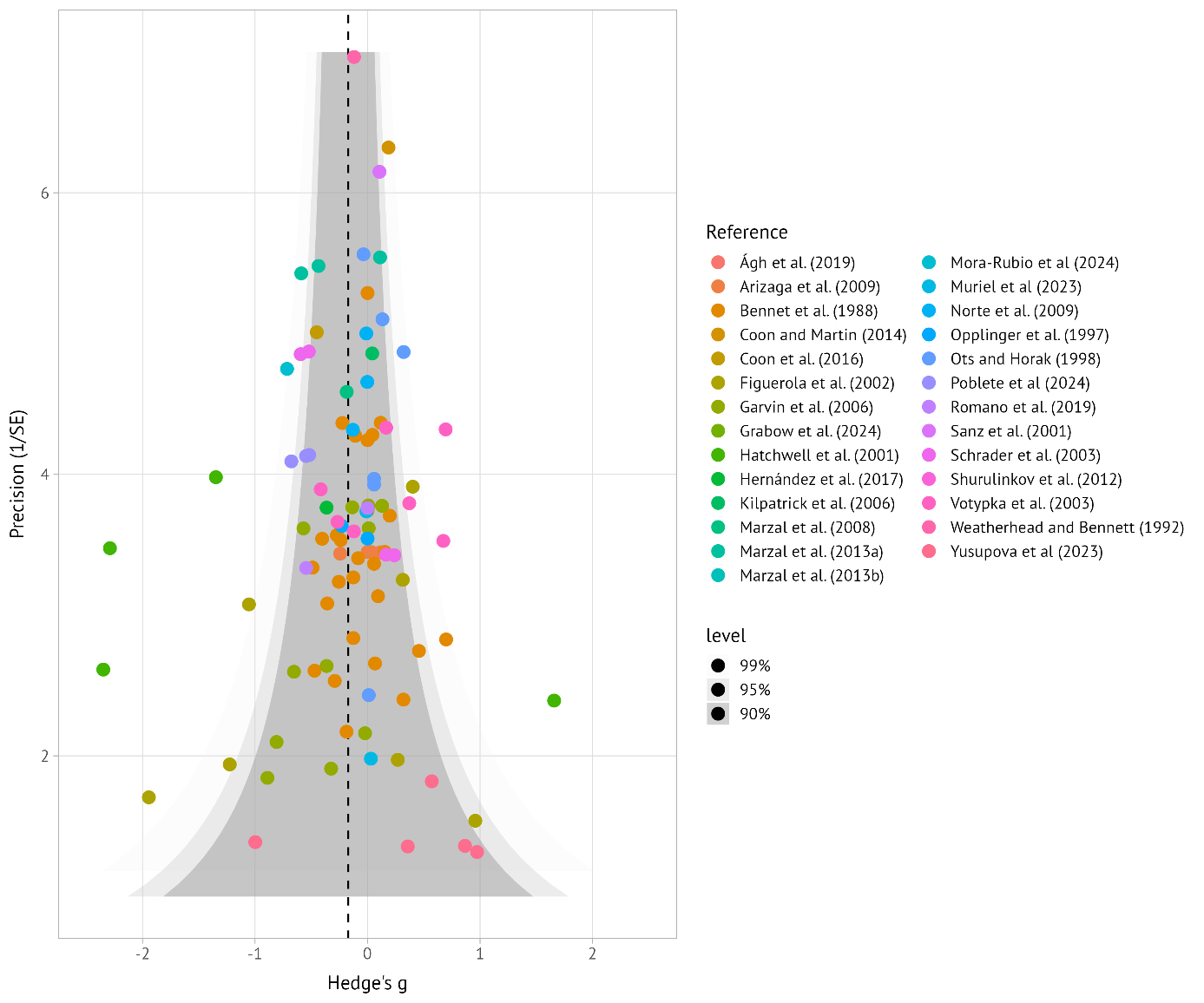


Figure S5: Funnel plot for host condition. Each point represents one effect size, coloured by study

## Survival


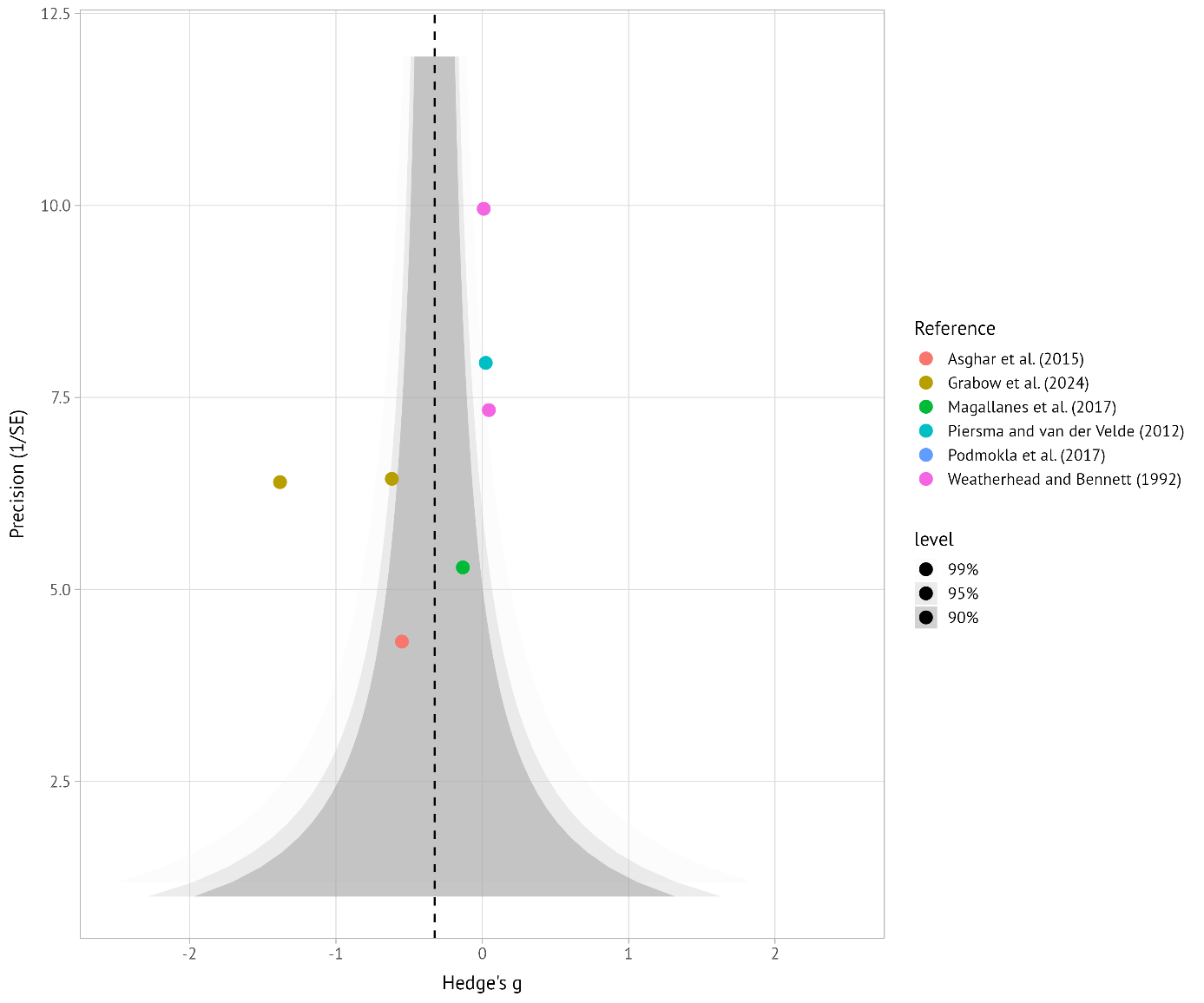


Figure S6: Funnel plot for host survival. Each point represents one effect size, coloured by study

## Reproduction


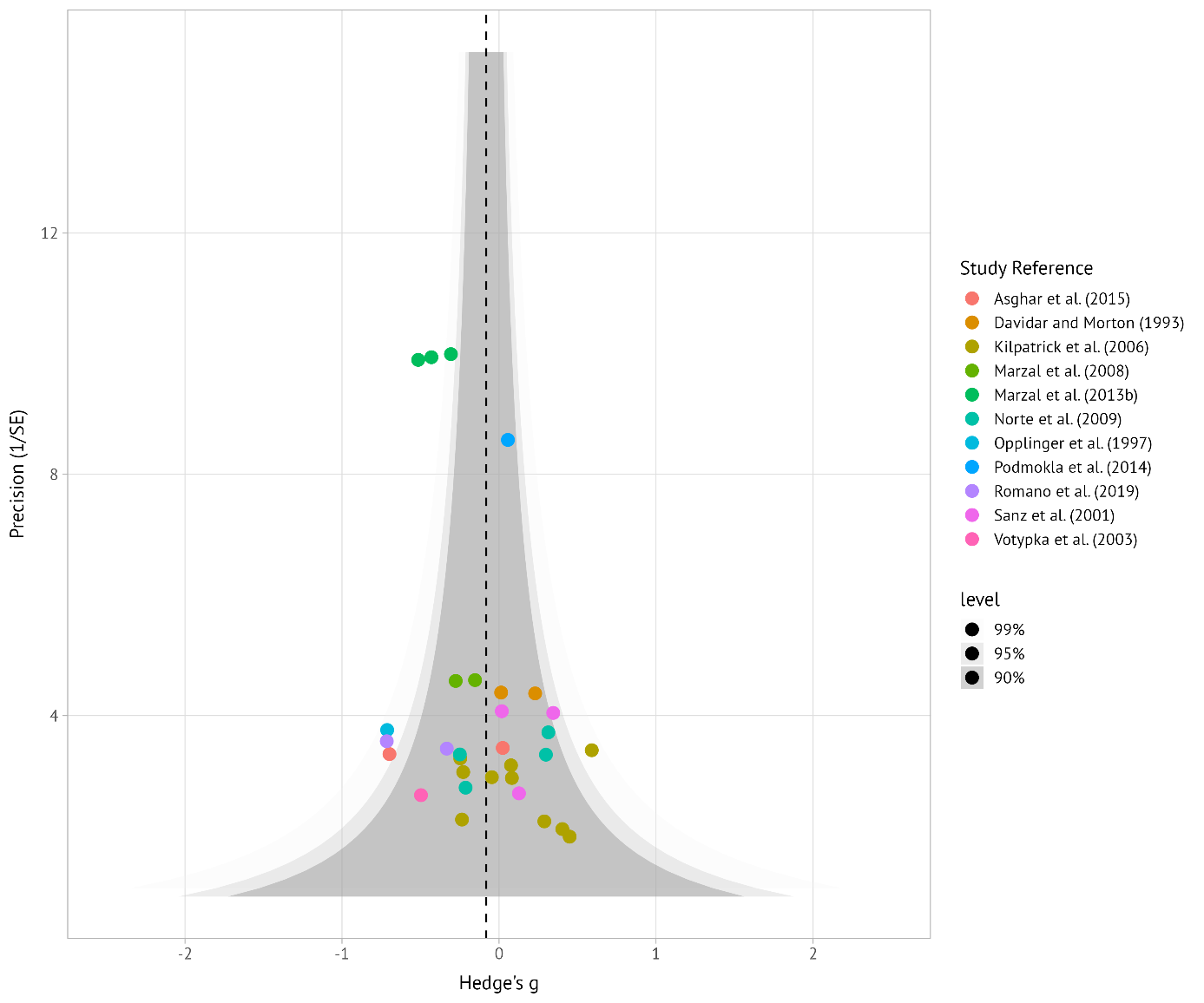


Figure S7: Funnel plot for host reproduction. Each point represents one effect size, coloured by study

## Phenology


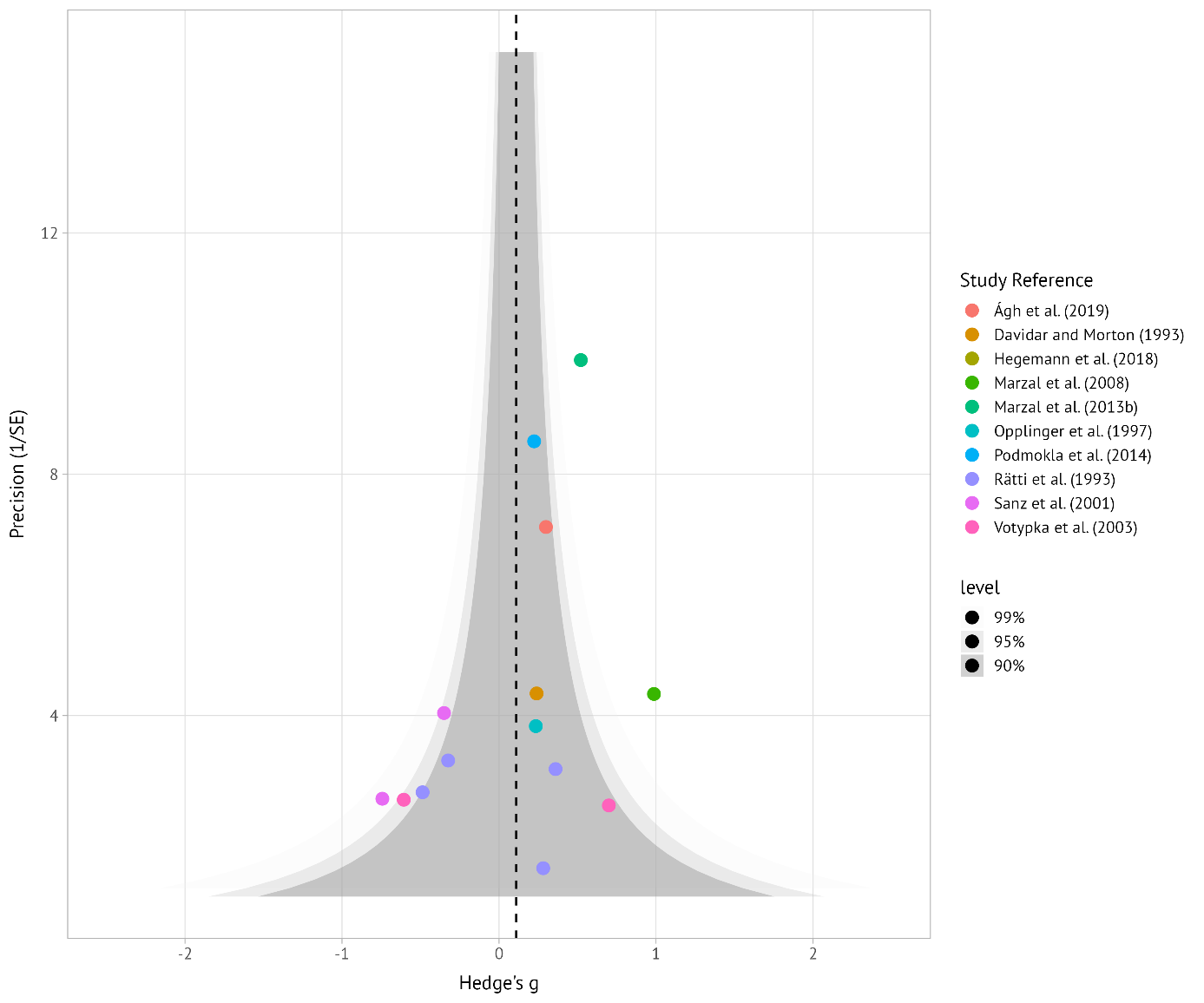


Figure S8: Funnel plot for phenology. Each point represents one effect size, coloured by study

# Effect size vs. effective sample size


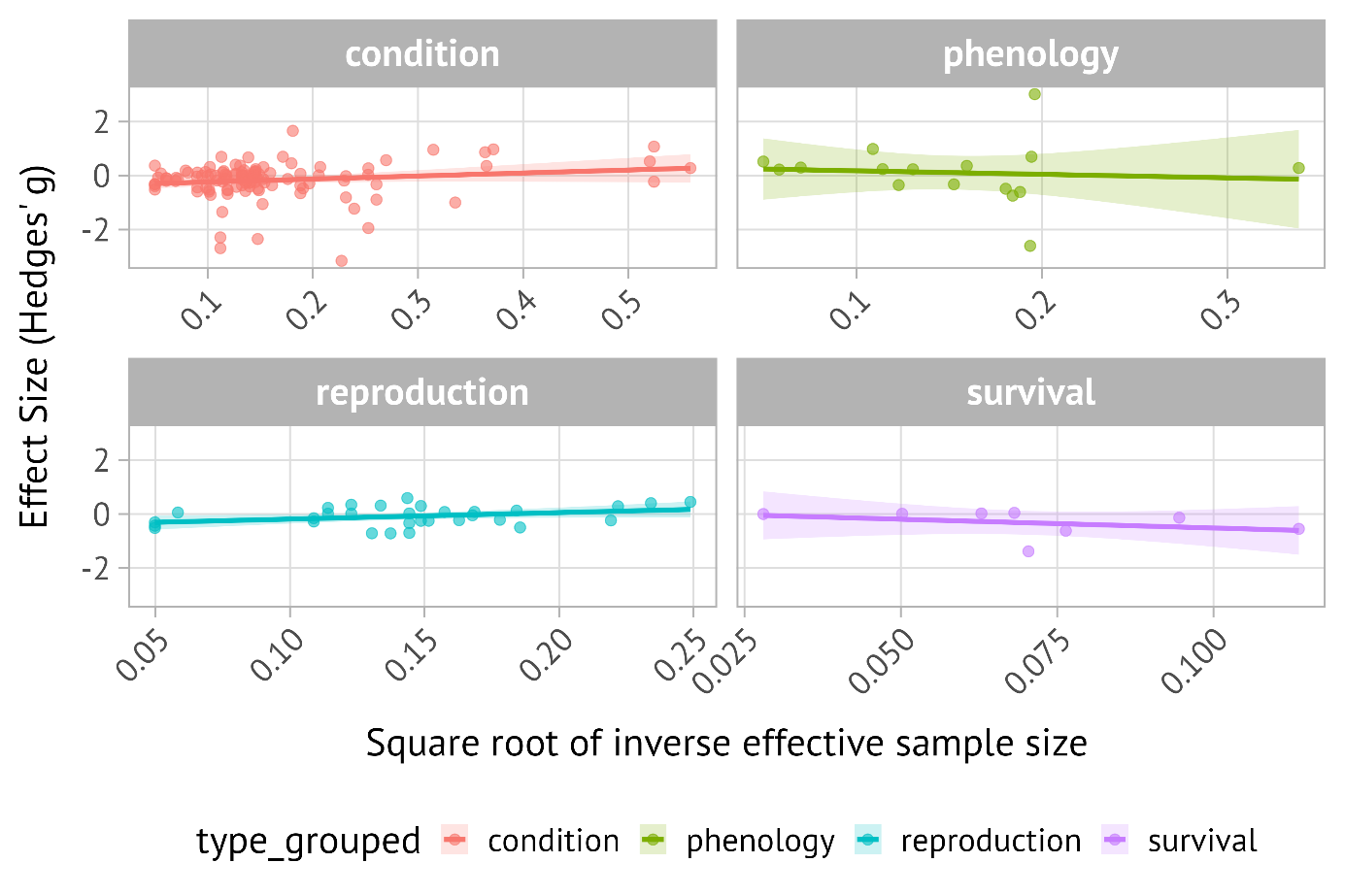


Figure S9: Relationship between inverse effective sample size and effect sizes. Each point represents an individual study, with colours differentiating study categories. Blue line and shaded are represent predicted effect size and 95%CI. The symmetrical horizontal distribution indicates minimal bias regarding study size.

# Effect size vs. study year


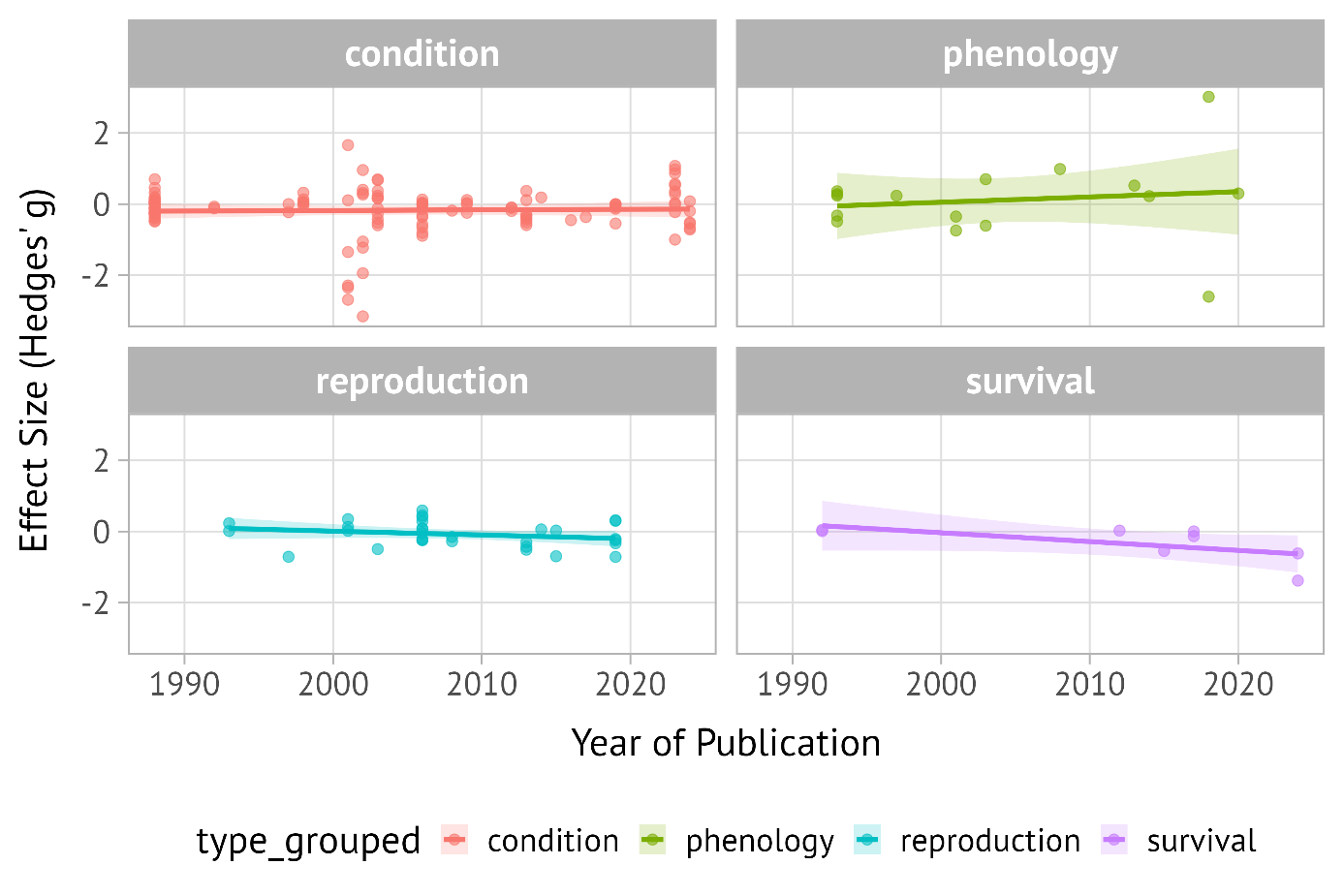


Figure S10: Relationship between study year and effect sizes. Each point represents an individual study, with colours differentiating study categories. Blue line and shaded are represent predicted effect size and 95%CI. The symmetrical horizontal distribution indicates minimal bias regarding study year.
